# Distinct neural signatures of phonemic and semantic verbal fluency: a double dissociation in cortical activation and functional connectivity revealed by fNIRS

**DOI:** 10.1117/1.NPh.13.2.025009

**Published:** 2026-05-12

**Authors:** Yize Cai, Yuexian Hou, Tian Zhu, Yanxin Yin, Chaolin Ma, Shilin Li, Weiming Sun

**Affiliations:** a The First Affiliated Hospital, Jiangxi Medical College, Nanchang University, Department of Rehabilitation Medicine, Nanchang, China; b Nanchang University, School of Public Policy and Management, Department of Psychology, Nanchang, China; c City University of Hong Kong, Department of Data Science, Hong Kong, China; d University of Hong Kong, Musketeers Foundation Institute of Data Science, Hong Kong, China; e Nanchang University, Jiangxi Medical College, The Second Affiliated Hospital, School of Basic Medical Sciences, Jiangxi Province Key Laboratory of Brain Science and Brain Health, Nanchang, China; f The First Affiliated Hospital, Jiangxi Medical College, Nanchang University, Postdoctoral Research Station, Nanchang, China

**Keywords:** verbal fluency task, phonemic fluency, semantic fluency, functional near-infrared spectroscopy, prefrontal cortex, functional connectivity

## Abstract

**Significance:**

The verbal fluency task (VFT) is a widely used paradigm in neuroimaging, yet its phonemic (pVFT) and semantic (sVFT) variants are frequently treated interchangeably despite limited understanding of their distinct neural substrates.

**Aim:**

We aim to systematically characterize the differences in cortical activation and functional connectivity (FC) patterns between pVFT and sVFT and to test the stability of these differences against individual variations in behavior and executive function, thereby providing actionable guidance for neuroscience research and clinical practice.

**Approach:**

We employed a within-subject design using functional near-infrared spectroscopy to compare cortical activation and FC during pVFT and sVFT. In-task behavioral performance and executive function measures were quantified to test whether individual differences could account for the neural dissociation.

**Results:**

We identified dissociated neural signatures between the two tasks. At the activation level, we observed that pVFT elicited significantly stronger activation across the prefrontal cortex (PFC), including the bilateral inferior frontal gyrus, dorsolateral prefrontal cortex, and orbitofrontal cortex. By contrast, sVFT was associated with greater activation in the bilateral supramarginal gyrus. At the network level, pVFT induced a state of higher global functional integration, characterized by enhanced long-range FC between PFC and posterior temporo-parietal regions. Critically, these neural dissociation patterns were not significantly modulated by variations in VFT behavioral performance or general executive function.

**Conclusions:**

pVFT and sVFT are subserved by partially nonoverlapping and stable neurocognitive systems. This has significant methodological implications, underscoring that the choice of VFT variant in future neuroscience research should be judiciously guided by the specific neural circuitry under investigation.

## 1 Introduction

Verbal fluency tasks (VFTs) are widely used in clinical neuroassessment and cognitive neuroscience.^1^^–^^4^ In a standard 60 s trial, participants are instructed to generate words according to either semantic or phonemic cues (sVFT, pVFT). In the sVFT, participants name as many exemplars as possible from a given semantic category (e.g., animals). In the pVFT, participants produce words beginning with a specified initial phoneme. Successful performance depends not only on lexical knowledge^5^ and retrieval abilities^6^ but also on executive control processes.^7^^–^^10^ These include working memory,^11^^–^^14^ cognitive flexibility, inhibition, processing speed, and attention.^15^ Because these functions are primarily supported by the prefrontal and temporal cortices, VFTs are considered a sensitive tool for probing the structural and functional integrity of these brain regions.^16^^–^^20^

Functional near-infrared spectroscopy (fNIRS) is portable, noninvasive, quiet, and relatively insensitive to motion artifacts, making it well suited for overt speech tasks such as VFT.^21^^,^^22^ fNIRS-VFT is now prominent in task-based studies. Our PubMed screen over the past year yielded 883 fNIRS papers (92 resting-state) and 56 using VFT, exceeding n-back (31) and comparable to Stroop (55), underscoring the centrality of VFT in task-based fNIRS studies.

Critically, pVFT and sVFT are not interchangeable. pVFT and sVFT differ markedly in cognitive and neural demands. pVFT relies more heavily on executive control processes, whereas sVFT places greater emphasis on language-related abilities.^8^^,^^9^^,^^23^^–^^26^ pVFT depends more on frontal systems, especially the left inferior frontal gyrus and broader dorsolateral prefrontal circuitry, which support retrieval strategies, switching, and inhibition.^27^^–^^29^ By contrast, sVFT emphasizes semantic/lexical systems engaging temporal cortices and temporo-parietal regions, supporting category-based retrieval and semantic clustering.^30^ Lesions to the temporal lobe selectively impair semantic fluency, whereas the prefrontal cortex affects both tasks.^3^^,^^28^^,^^31^^,^^32^

However, fNIRS studies frequently treat the two tasks as a single paradigm or omit justification for task choice. Of the 56 VFT-fNIRS papers we reviewed, four did not specify task type; 34 used only pVFT, 15 only sVFT, and only three employed both. Two studies on nonsuicidal self-injury (NSSI) selected different VFT types without providing a rationale and reported differing activation patterns,^33^^,^^34^ which highlights a potential concern: failing to distinguish or justify the use of different VFT types within a single study may obscure the interpretation of neural mechanisms underlying brain activation, thereby limiting reproducibility and comparability across studies. Where both tasks were included, disorder-specific dissociations emerged: Li et al.^35^ reported widespread cortical activation in schizophrenia across both pVFT and sVFT, but more limited, sVFT-specific activation in bipolar I (BD-I), implying task-dependent sensitivity to clinical phenotypes, which could serve as a potential auxiliary diagnostic tool. Duan et al.^30^ explicitly compared task sensitivity before data collection and then chose sVFT, illustrating how pre-task considerations can shape design. These studies collectively point to a critical gap in systematically comparing the neurolinguistic and neurophysiological implications of pVFT and sVFT.

To provide methodological guidance for future fNIRS studies and clinical applications, systematic, within-subject comparisons of pVFT and sVFT are urgently needed. We identify two specific gaps: First, no study has conducted a fine-grained, within-subject region of interest (ROI) activation contrast of pVFT versus sVFT; parietal regions remain largely unexamined, and ROI-level lateralization has not been systematically addressed. Moreover, although 10/56 VFT–fNIRS studies last year assessed task-evoked functional connectivity (FC), none conducted a multiscale FC comparison between tasks. Second, whether individual differences in fluency behavior and executive function—given that pVFT likely relies more on executive processes—modulating the activation differences between pVFT and sVFT remains unclear. Multidimensional behavioral measures [e.g., correct responses (CRs), response time dynamics, clustering, and switching indices] are powerful tools for capturing individual VFT performance,^1^^,^^2^ yet these measures have not been incorporated into fNIRS–VFT research.

The present study addresses these gaps using a within-subject design in healthy adults, in which both pVFT and sVFT were administered during fNIRS. We conducted ROI-level activation contrasts and a multiscale comparison of FC between tasks and tested whether neural differences are related to individual VFT performance and are modulated by executive abilities assessed in this study.

This work provides methodological guidance for selecting and interpreting fNIRS tasks, thereby enhancing consistency and comparability across studies. In addition, by clarifying the differential sensitivity of pVFT and sVFT, our findings have important implications for clinical assessment and monitoring. Ultimately, this study delineates distinct neural signatures for each task, which advances our understanding of how executive and semantic systems are differentially engaged in verbal fluency. Building on these aims, the following section outlines the participants and methodology employed in our research.

## 2 Method

### 2.1 Participants

Eighty-one right-handed participants (35 males, 46 females; mean age=23.6±2.3 years) were recruited through posters and online advertisements. All participants had normal or corrected-to-normal visual acuity, with no color blindness and no known neurological disorders. To ensure compliance with task demands, particularly in the phonemic fluency condition, which relies on the use of Mandarin Pinyin, participants were required to demonstrate full proficiency in the Pinyin system. All participants provided written informed consent. The experimental procedures were approved by the Ethics Committee of the First Affiliated Hospital of Nanchang University (Ethics Number: IIT2025 Clinical Ethics Review No. 642). After excluding three participants due to low-quality fNIRS data, the final sample consisted of 78 participants.

### 2.2 Procedure

**Fig. 1:**
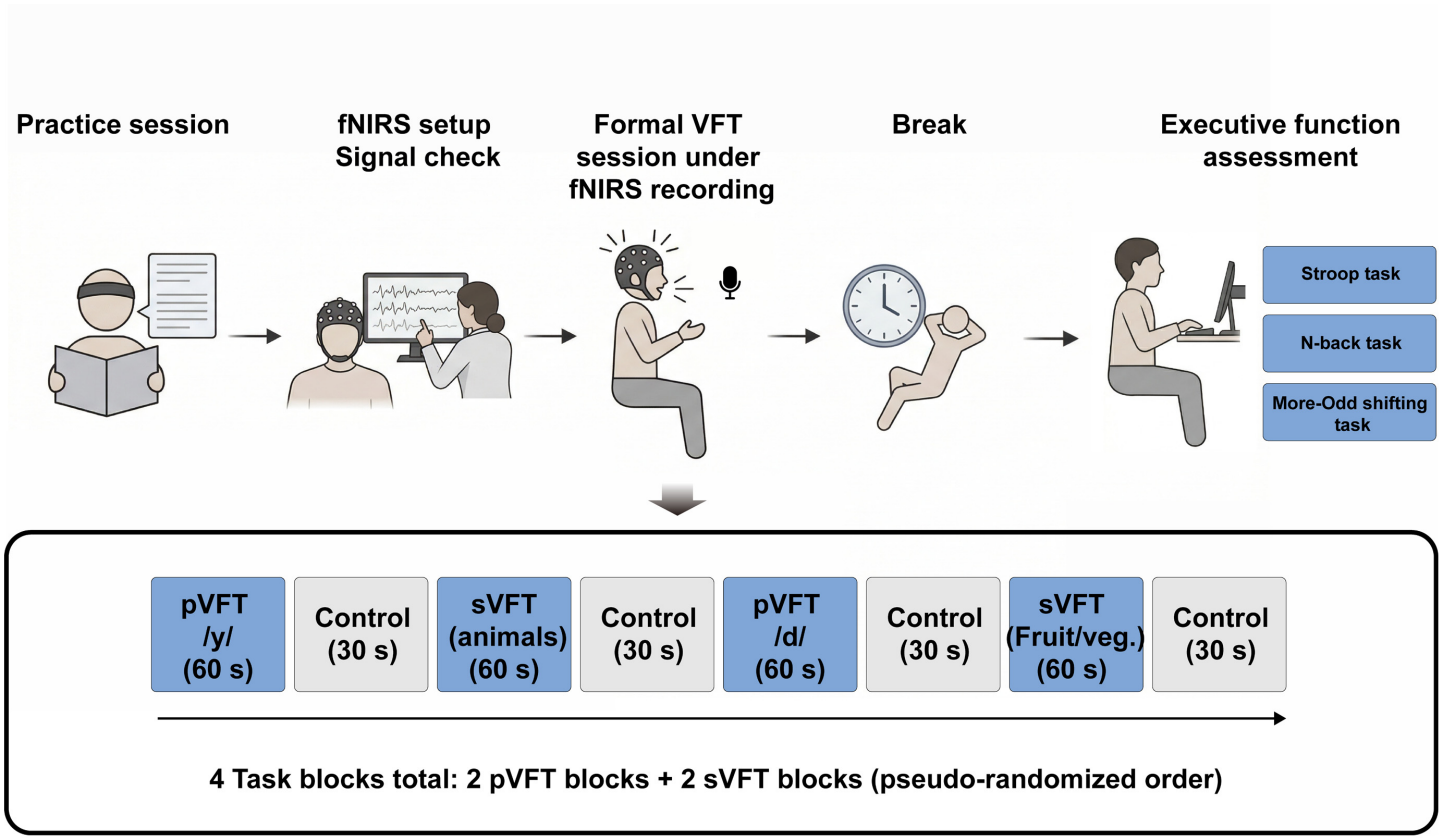
Schematic illustration of the experimental procedure. After a brief practice session, participants underwent fNIRS cap placement and signal quality check. They then completed the formal VFT session during continuous fNIRS recording, followed by a short break and a separate executive function assessment battery comprising the Stroop task, N-back task, and More–Odd Shifting task. The VFT session consisted of four 60-s task blocks and four 30-s control blocks, including two pVFT blocks using the Mandarin Pinyin initial consonants /y/ and /d/, and two sVFT blocks using the categories animals and fruits/vegetables. The task blocks were presented in a pseudo-randomized order, and the sequence shown here is for illustrative purposes.

All participants were tested individually in a quiet, dimly lit experimental room. Before the formal task, each participant completed a brief practice session to become familiar with the VFT procedure. Following the practice, a standard fNIRS head cap was positioned according to the international 10–20 system. During setup, the signal quality of each channel was monitored in real time using the acquisition software. If any suboptimal signal quality was detected, adjustments were made either by repositioning the cap or by gently parting the hair to improve optode–scalp contact. Once optimal signal quality was confirmed, participants were seated in a comfortable upright position. To ensure consistency and reduce movement artifacts, participants were instructed to rest their upper right forearm on the table and minimize head and body movements throughout the recording session. All stimuli were programmed and presented using E-Prime 2.0 software (Psychology Software Tools, Sharpsburg, Pennsylvania, United States). After the setup was completed, participants first performed the formal VFT session while fNIRS signals were continuously recorded. Following the VFT session, participants were given a short break and then completed a separate executive function assessment battery, including the Stroop task, N-back task, and More–Odd Shifting task (Fig. 1).

### 2.3 Behavioral Tasks

#### 2.3.1 Verbal fluency task

The VFT consisted of two pVFT blocks and two sVFT blocks, each lasting 60 s, which were presented in a pseudo-randomized order. A 30 s control block followed each task block. For the sVFT, participants were instructed to generate as many words as possible within two designated semantic categories: “animals” and “fruits or vegetables.” For the pVFT, participants were instructed to produce multicharacter Chinese words beginning with the Mandarin Pinyin initial consonant /y/ in one block and /d/ in the other block, which were selected because they are among the initials with the largest word-forming capacity in common usage. This paradigm closely resembles the letter fluency tasks used in Indo-European languages as it relies on the phonemic properties of Pinyin rather than the orthographic features of Chinese characters. Accordingly, it is regarded as a pure phonemic task, primarily engaging phonological retrieval mechanisms.^36^ During the control blocks, participants were instructed to count from 1 to 5 continuously in a loop. To minimize variability in vocal output, the counting speed was calibrated to approximately one number every 2 s.

Participants’ verbal responses were recorded using a microphone for subsequent behavioral analysis. Each naming response was time-stamped using Praat,^37^ and the annotated onset of each spoken word served to extract a series of behavioral variables. The definitions and extraction procedures for these variables were based on the framework described by Bose et al.^1^ A summary of these variables is provided in Table 1.

**Table 1:** Behavioral variables extracted from the verbal fluency task, based on Bose et al.^1^

Parameter	Description
Number of correct responses (CRs)	Number of valid, nonrepetitive, and task-appropriate words generated in each 60 s block. Reflects general word retrieval ability.
Sub-RT (response time)	Mean interval between the onset of successive responses (excluding first). Estimates retrieval latency and reflects retrieval dynamics.
Average cluster size	Average number of words produced within the same semantic or phonemic subcategory. Reflects access to lexical networks.
Number of switches	Strategic process to shift efficiently to a new subcategory when a subcategory is exhausted
Within-cluster pauses (WCP)	Mean time differences between each successive word within the same cluster
Between-cluster pauses (BCP)	Mean silent interval between the last word of one cluster and the first word of the next.

#### 2.3.2 Clustering and switching criteria

Errors were excluded from the calculation of correct responses (CR). In semantic fluency, errors included repetitions, nonstandard words, and category-inappropriate items. In phonemic fluency, errors included repetitions, nonstandard words, unintelligible utterances, words beginning with an incorrect initial consonant, and proper nouns. All criteria were explicitly explained to participants in advance to minimize violations. Although repetition errors were excluded from CR counts, they were retained for clustering and switching analyses because they are thought to reflect underlying cognitive organization regardless of their inclusion in the total word count.^2^

Cluster size was calculated from the second word onward within each cluster. A single ungrouped word (e.g., “dog”) was coded as cluster size 0; two semantically related words (e.g., “cat–dog”) were coded as size 1, and so on. Switching was defined as transitions between clusters. For instance, if a participant said “whale–dolphin” (marine animals), then “eagle–hawk” (wild birds), and then “chicken–duck” (domestic animals), it was counted as two switches: from marine animals to wild birds and then from wild birds to domestic animals.

Clustering criteria were adapted from Troyer et al.^2^ and refined to suit the linguistic and cultural features of Mandarin Chinese. Specifically, phonemic clusters were defined based on shared initial consonants or syllables in Pinyin, with adjustments to accommodate tonal distinctions and morphological characteristics specific to Mandarin. Two independent raters evaluated clustering and switching patterns, and any discrepancies were resolved through discussion.

#### 2.3.3 Executive function tasks

Executive function, defined by Miyake et al.^38^ as three separable subcomponents (shifting, updating, and inhibition), was evaluated using the More–Odd Shifting task,^39^ the N-back task,^40^ and the Stroop task.^41^ Detailed task designs are described in Sec. 1 of the Supplementary Material. For the Stroop task (inhibition), the outcome measure was the Stroop ratio, defined as the Stroop difference (mean RT incongruent minus mean RT neutral) divided by the mean of the two conditions, with smaller values indicating better inhibitory control. For the N-back task (updating), the outcome measure was accuracy, defined as the proportion of correct responses to target stimuli. For the More–Odd Shifting task (shifting), the outcome measure was the shifting cost, defined as the RT difference between the shifting and nonshifting conditions.

### 2.4 fNIRS data acquisition

fNIRS data were continuously recorded throughout the pVFT and sVFT with a continuous-wave near-infrared spectrometer (NirSmart-6000A, Danyang Huichuang Medical Equipment Co., Ltd., China). The system employs LED light sources and avalanche photodiodes (APDs) as detectors, operating at wavelengths of 730 and 850 nm with a sampling rate of 11 Hz. Optode placement followed the international 10–20 system. The fNIRS cap consisted of 21 sources and 16 detectors, forming 48 channels with a fixed inter-optode distance of 3 cm. Of these, 40 channels covered the bilateral prefrontal and temporal cortices, and the remaining eight channels targeted the bilateral supramarginal gyrus (SMG) in the parietal cortex.

#### 2.4.1 fNIRS optode locations

The montage was designed to maximize coverage of the PFC and temporal lobes, regions consistently implicated in verbal fluency performance. Both pVFT and sVFT rely on the PFC, with pVFT particularly dependent on the left inferior frontal gyrus.^3^^,^^27^^,^^28^ sVFT performance has been linked to semantic representations in the temporal lobe, especially the left hemisphere,^27^^,^^31^^,^^32^ and neuroimaging evidence shows that both task types elicit robust activation in frontotemporal cortices.^42^^,^^43^

**Fig. 2:**
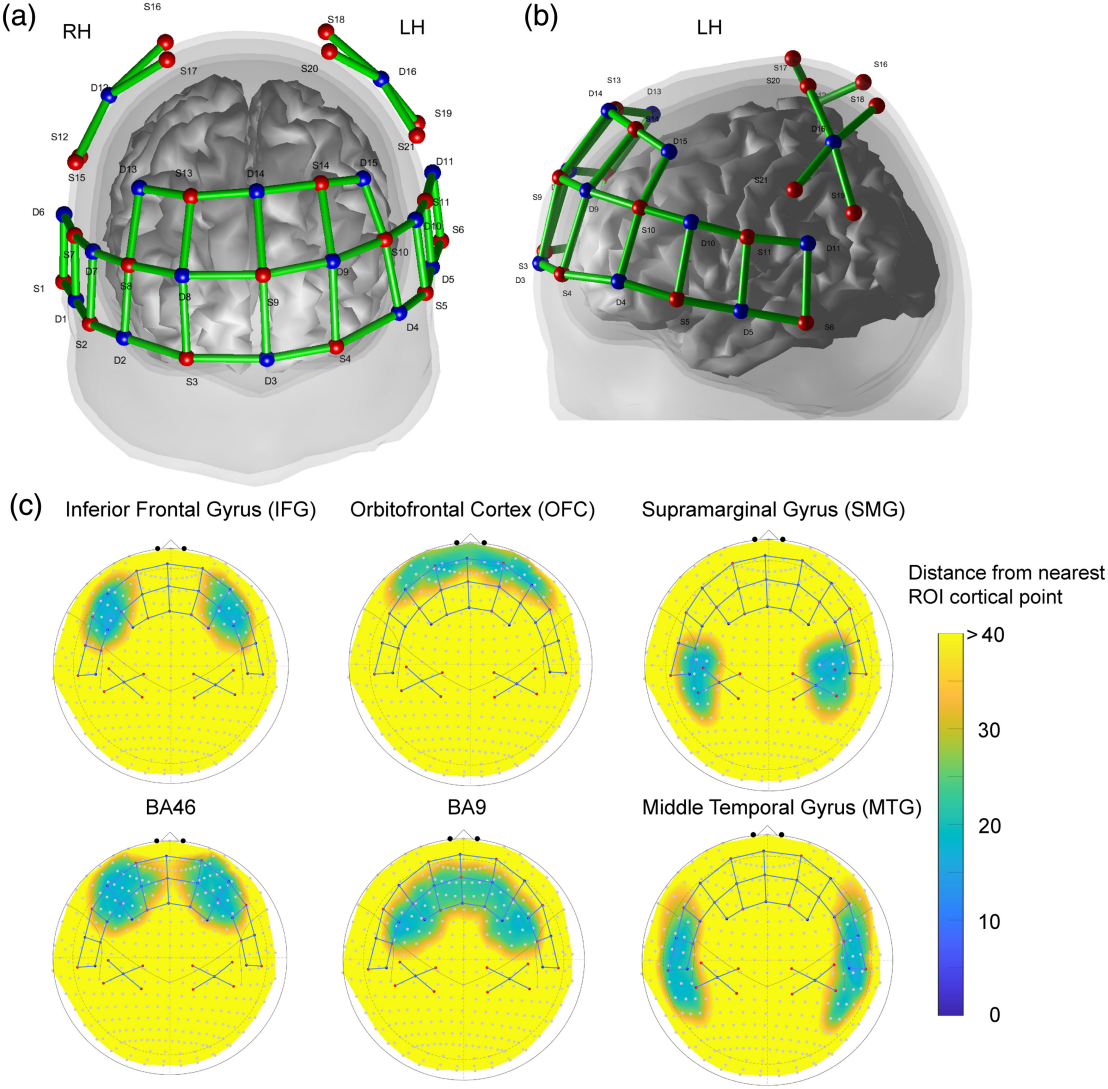
fNIRS montage and ROI setup. (a) Montage in frontal view. (b) Montage in left-hemisphere view. The montage included 21 sources and 16 detectors, forming 48 channels at a fixed inter-optode distance of 3 cm. Of these, 40 channels covered the bilateral prefrontal and temporal cortices, and eight channels targeted the bilateral SMG in the parietal cortex. Sources are shown in red, detectors in blue, and channels in green lines. (c) Gross ROI depth maps with superimposed montage in the international 10–20 coordinate space. Six ROIs were identified: IFG, DLPFC (including BA9 and BA46), OFC, SMG, and MTG. Channels covering blue and green areas are within range of the ROI; channels over yellow and orange areas are outside of the ROI.

The parietal cortex, particularly the SMG, has also been associated with deficits in both pVFT and sVFT following lesions.^28^^,^^29^^,^^32^ The SMG is a key component of the speech–language network^44^ and is considered to function as a phonological store in working memory models.^45^ Therefore, the parietal region was included as an additional ROI. The fNIRS optode layout is illustrated in Figs. 2(a) and 2(b).

The gross anatomical locations of the ROIs in the montage were determined using the depth map function of the Brain AnalyzIR Toolbox.^46^ Depth maps estimate the distance from each fNIRS optode to the superficial cortex of various Talairach Daemon-labeled regions within the Colin27 atlas.^47^^,^^48^ Channels that project onto yellow or orange areas in Fig. 2(c) indicate a depth greater than 30 mm and thus are unlikely to reach the specified ROI. By contrast, channels overlying green or blue regions are within the effective penetration range of the nearest cortical surface within the targeted ROI. Based on this information, we defined six ROIs in each hemisphere: the inferior frontal gyrus (IFG), the dorsolateral prefrontal cortex (DLPFC; including Brodmann Area (BA)9 and BA46), the orbitofrontal cortex (OFC), the SMG, and the middle temporal gyrus (MTG). The specific source-detector pairs for each ROI are listed in Data 1 of the Supplementary Material.

### 2.5 Analysis

#### 2.5.1 Behavioral data analysis

Executive function task data were extracted from E-Prime 2.0, and VFT responses were time-stamped in Praat, and all analyses were conducted in R 4.3.1. To evaluate the validity of the executive function tasks, statistical analyses were conducted on reaction times (RTs) and accuracy from the Stroop, N-back, and More–Odd Shifting tasks. For the Stroop task, a paired-samples t-test was used to compare RT and accuracy between the two conditions (neutral versus interference). Subsequently, one-way repeated-measures ANOVA was performed on RT and accuracy for the N-back task (0b, 1b, 2b conditions) and the More–Odd Shifting task (More, Odd, Shifting conditions), followed by Bonferroni-corrected post hoc tests. VFT behavioral variables (Table 1) were computed after excluding incorrect responses and compared between pVFT and sVFT using paired-samples t-tests. When normality was violated, the Wilcoxon signed-rank test was used.

#### 2.5.2 fNIRS data analysis

##### Quality control of the fNIRS data

Raw intensity was visually inspected in NirSpark (Danyang Huichuang Medical Equipment Co., Ltd., China) to verify the presence of cardiac oscillations as an indicator of data quality.^49^ Channels with a coefficient of variation greater than 7.5% were considered physiologically implausible and thus flagged as invalid^49^; participants with more than 15% invalid channels were excluded from further analysis. All participants passed the first step, whereas three were excluded in the second step.

##### fNIRS data pre-processing pipeline

Data were processed in MATLAB R2021b (MathWorks Inc., Massachusetts, United States) using the Brain AnalyzIR Toolbox.^46^ Data were truncated 30 s before task onset and 30 s after task offset, and intensity signals were converted to optical density and subsequently to oxyhemoglobin (HbO) and deoxyhemoglobin (HbR) concentrations via the modified Beer–Lambert law.^50^ For FC, optical density signals were low-pass filtered at 0.4 Hz.

##### Analysis of task-based activation

Task-based activation was analyzed using a general linear model (GLM) pipeline with iteratively reweighted least-squares (AR-IRLS) estimation, which accounts for serially correlated errors and down-weights motion-related outliers.^51^ At the group level, β estimates were entered into mixed-effects models with condition as a fixed effect and subject as a random effect,^46^ and significance was determined using FDR correction. Detailed descriptions of the GLM steps are provided in Sec. 2 of the Supplementary Material.

Overall, the HbR findings were broadly consistent with the HbO results, although weaker in magnitude and less spatially extensive. Given that HbO provides a higher signal-to-noise ratio and greater spatial sensitivity in fNIRS studies, HbO was retained as the primary outcome measure, and HbR is presented as complementary convergent evidence. HbR results are reported throughout the text and in the Supplementary Material, including Figs. S1–S4 and the corresponding Data sections in the Supplementary Material.

##### Contrasting task-based activation of pVFT and sVFT

To examine activation differences between pVFT and sVFT at the ROI level and to assess hemispheric lateralization, a three-way repeated-measures ANOVA was conducted with ROI, hemisphere, and task as within-subject factors. In addition, three sets of linear mixed-effects (LME) analyses were performed: the first tested condition differences in activation within each ROI, the second quantified lateralization effects by contrasting left and right hemispheric activations, and the third compared lateralization between the two tasks across ROIs. These analyses were designed to capture complementary aspects of task-related activation and lateralization. The ANOVA and LME specifications are provided in Sec. 2 in the Supplementary Material.

##### Dynamic activation analysis of VFT

To investigate the dynamic activation differences between the two VFTs, task blocks were truncated to 10, 20, 30, and 50 s from task onset. A separate GLM was constructed for each of these time windows to capture the temporal evolution of task-related activation. This dynamic GLM approach was adapted from Mukli et al.^52^ Consistent with our analysis of the whole task block, dynamic contrasts between pVFT and sVFT were obtained for each of these time windows.

##### Analysis of functional connectivity

Functional connectivity (FC) was estimated using AR-whitened Pearson correlations to reduce physiological noise and temporal autocorrelation. Correlation values were Fisher-transformed, averaged within condition, and back-transformed to obtain a single FC value per participant per task. Group-level connectivity was then analyzed using linear mixed-effects models, which yielded adjacency matrices for pVFT, sVFT, and their contrast. FDR correction was applied to identify significant connections. To further characterize network topology, graph-theoretical measures were computed. Binary node degrees captured the number of significant connections. By contrast, weighted node degrees quantified their strength, both at local and global levels. Detailed descriptions of FC estimation, thresholding, and graph analyses are provided in Sec. 2 in the Supplementary Material.

##### Contrasting functional connectivity of pVFT and sVFT

To examine differences in functional connectivity between the two fluency tasks, a series of LME analyses was conducted using averaged Fisher Z-transformed connectivity within each connection type as the dependent variable. Connection types were classified hierarchically into hemispheric (intra-left, intra-right, inter-hemispheric), within-ROI, and inter-ROI categories, with a separate LME applied for each using the formula “Z∼ Condition × ConnectionType + (1 | Subject).” Post hoc comparisons were performed using the emmeans package to estimate the simple effects of Condition within each connection type, with a focus on the contrast between phonemic and semantic conditions.

In addition to connection-level analyses, graph-theoretical metrics were examined to evaluate differences in the number and strength of node connections between tasks. For local node degrees (weighted and binarized), channel-level LMEs were conducted to derive condition-specific β values and direct contrasts, which were then mapped onto the 10–20 system. Global node degrees were compared between tasks using paired-samples t-tests on individual-level weighted and binarized measures. Detailed descriptions of these analyses are provided in Sec. 2 in the Supplementary Material.

#### 2.5.3 Assessing the Influence of Individual Differences on Neural Patterns

To examine whether the task-related activation patterns were modulated by the specific individual differences assessed in this study, a series of LME models was performed. First, to examine the influence of in-task behavioral performance, models were constructed for each behavioral variable and ROI using the formula “Activation ∼ Condition * Behavioral_Performance + (1|Subject).” We extracted the main effect of the behavioral variable and its interaction with the condition factor. Second, to assess the influence of general executive functions, analogous LME models were constructed for each of the three executive function measures using the formula “Activation ∼ Condition * Executive_Function_Score + (1|Subject).” Executive function measures were operationalized as the Stroop ratio for inhibition, 2-back accuracy for updating, and the shifting cost for shifting.

## 3 Results

### 3.1 Bilateral PFC Activation in pVFT and Bilateral SMG Recruitment in sVFT

**Fig. 3:**
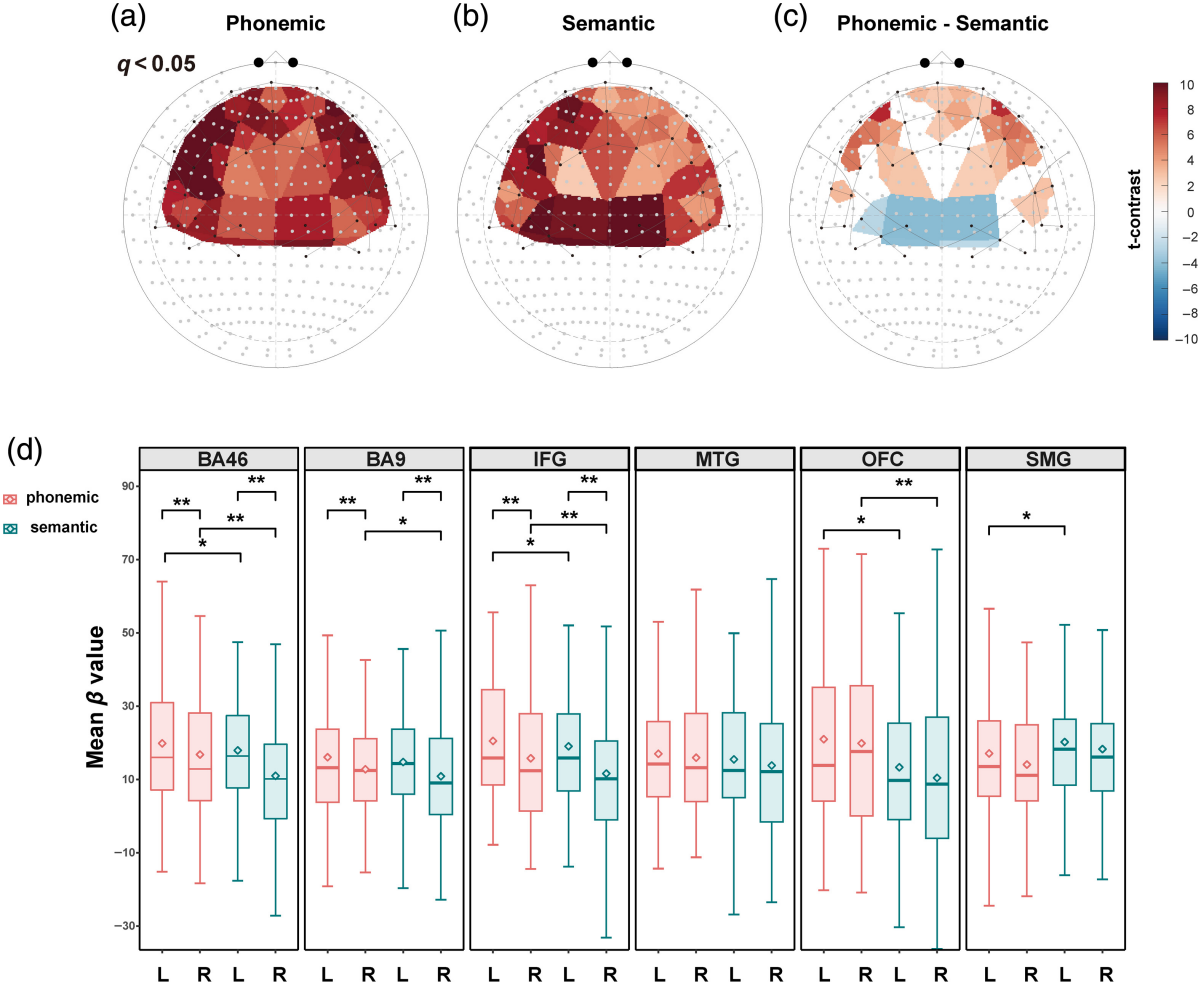
Activation statistics at the channel and ROI levels. (a)–(c) FDR-corrected t-statistics (indicated by the color-coded t-values with a cutoff at q<0.05) for the task-specific HbO activation patterns of pVFT, sVFT, and the contrasts between pVFT and sVFT. (d) Boxplots of mean HbO beta values within each ROI for pVFT and sVFT. Diamonds represent mean values, and significance levels derived from the LME contrasts are indicated on the plots (*p<0.05, **p<0.01).

Task-specific brain activation patterns were assessed by analyzing changes in oxyhemoglobin (HbO) levels during the two VFTs. At the channel level, Figs. 3(a)–3(c) show the FDR-corrected t-statistics (q<0.05) for both VFTs as well as their contrasts. A detailed summary of the β values, p-values, q-values, and statistical power for all channels is provided in Data S2 in the Supplementary Material.

The pVFT elicited widespread HbO increases across the probe array, with the strongest effects observed in the bilateral PFC, particularly the left IFG, most of the left DLPFC, and the anterior portion of the left temporal lobe. By contrast, sVFT activation was more restricted, with pronounced responses limited to a few channels in the left IFG and left DLPFC, along with marked activation in the bilateral SMG.

In the contrast map, pVFT elicited significantly stronger activation across large portions of the bilateral PFC, especially in the left IFG, left DLPFC, and several right DLPFC channels. Minor differences were observed in the MTG, confined to three channels, whereas the bilateral SMG showed relatively greater activation during sVFT.

HbR changes were generally weaker than HbO and less spatially extensive. HbR maps of the single task showed limited effects for both pVFT and sVFT. The contrast map revealed a task-dependent spatial pattern consistent with the HbO results, with greater pVFT effects in portions of the bilateral IFG and BA46 and greater sVFT effects in the bilateral SMG [Fig. S1(A)–S1(C) in the Supplementary Material].

### 3.2 Task-ROI Dissociation with Overall Leftward Dominance

A three-way repeated-measure ANOVA was conducted on the ROI-level mean β values with task (phonemic versus semantic), ROI, and hemisphere (left versus right) as within-subject factors. A significant main effect of hemisphere was observed, F(1,77)=23.11, p<0.001, indicating that overall activation was greater in the left hemisphere than in the right. No significant main effect of task or ROI was found.

Importantly, a significant task × ROI interaction was detected, F(5,385)=8.26, p<0.001, suggesting that the activation difference between phonemic and semantic tasks varied across ROIs. A significant ROI × hemisphere interaction was also observed, F(5,385)=3.07, p=0.010, indicating that lateralization patterns differed by ROI. All other interactions, including task × hemisphere and the three-way interaction of task × ROI × hemisphere, were nonsignificant. For HbR, the repeated-measure ANOVA similarly revealed a significant task × ROI interaction, F(5,385)=2.54, p=0.028, along with a significant main effect of ROI, F(5,385)=3.23, p=0.007. In contrast to HbO, neither the main effect of hemisphere nor any other interaction reached significance. These results suggest that, although weaker overall, HbR responses also showed task-dependent regional dissociation across ROIs. Sphericity violations were detected for several factors (Mauchly’s tests, ps<0.01), and Greenhouse–Geisser corrections were applied where appropriate.

#### 3.2.1 pVFT stronger in the PFC, sVFT stronger in the SMG, and MTG no difference

To further examine activation differences between pVFT and sVFT within each ROI, ROI-wise t-contrasts were conducted using subject-level averaged β values. Significant differences were observed in multiple frontal regions, with stronger activation during pVFT than sVFT. In all prefrontal regions except the left BA9, pVFT activation was significantly greater than that during sVFT (ps<0.05).

Notably, sVFT elicited greater activation than pVFT in the SMG (L-SMG: β=−3.63, p=0.033; R-SMG: β=−3.36, p=0.053). This pattern diverged from the predominant pVFT > sVFT trend observed across other regions and was consistent with the channel-wise contrast maps. No significant differences were found in the bilateral MTG. These results are displayed in Fig. 3(d), which shows the mean activation values in each ROI across hemispheres and conditions.

ROI-wise HbR comparisons showed significant task differences in the right IFG and bilateral SMG (R-IFG: β=−2.43, p=0.016; L-SMG: β=2.86, p=0.025; R-SMG: β=2.76, p=0.021). Consistent with the HbO results, pVFT showed relatively greater effects in the right IFG, whereas sVFT showed relatively greater effects in the bilateral SMG. No significant differences were found in the other ROIs [Fig. S1(D) in the Supplementary Material].

#### 3.2.2 HbO showed leftward dominance in IFG/BA46/BA9 under both tasks

To evaluate hemispheric lateralization, left and right activations were compared within each ROI under both conditions. Significant leftward lateralization was observed in the IFG (pVFT: β=4.28, p<0.001; sVFT: β=4.48, p<0.001), BA46 (pVFT: β=4.01, p<0.001; sVFT: β=5.14, p<0.001), and BA9 (pVFT: β=2.00, p=0.001; sVFT: β=2.94, p<0.001), indicating a consistent pattern of leftward dominance across frontal regions. These results are illustrated in Fig. 3(d).

HbR did not exhibit significant hemispheric asymmetry across any of the ROIs [Fig. S1(D) in the Supplementary Material]. This lack of lateralization is potentially attributable to its generally attenuated and less spatially distributed response pattern.

#### 3.2.3 HbO showed greater left-lateralization for sVFT than pVFT in IFG and SMG

Comparison of hemispheric lateralization between the phonemic and semantic fluency tasks revealed significantly greater left-lateralization in two regions during the semantic task compared with the phonemic task: IFG (β=−3.09, p<0.001) and SMG (β=−1.44, p=0.034). For HbR, greater left-lateralization for sVFT than pVFT was observed in the SMG (β=2.12, p=0.002) and MTG (β=2.81, p<0.001).

### 3.3 Dynamic brain activation during VFT

**Fig. 4:**
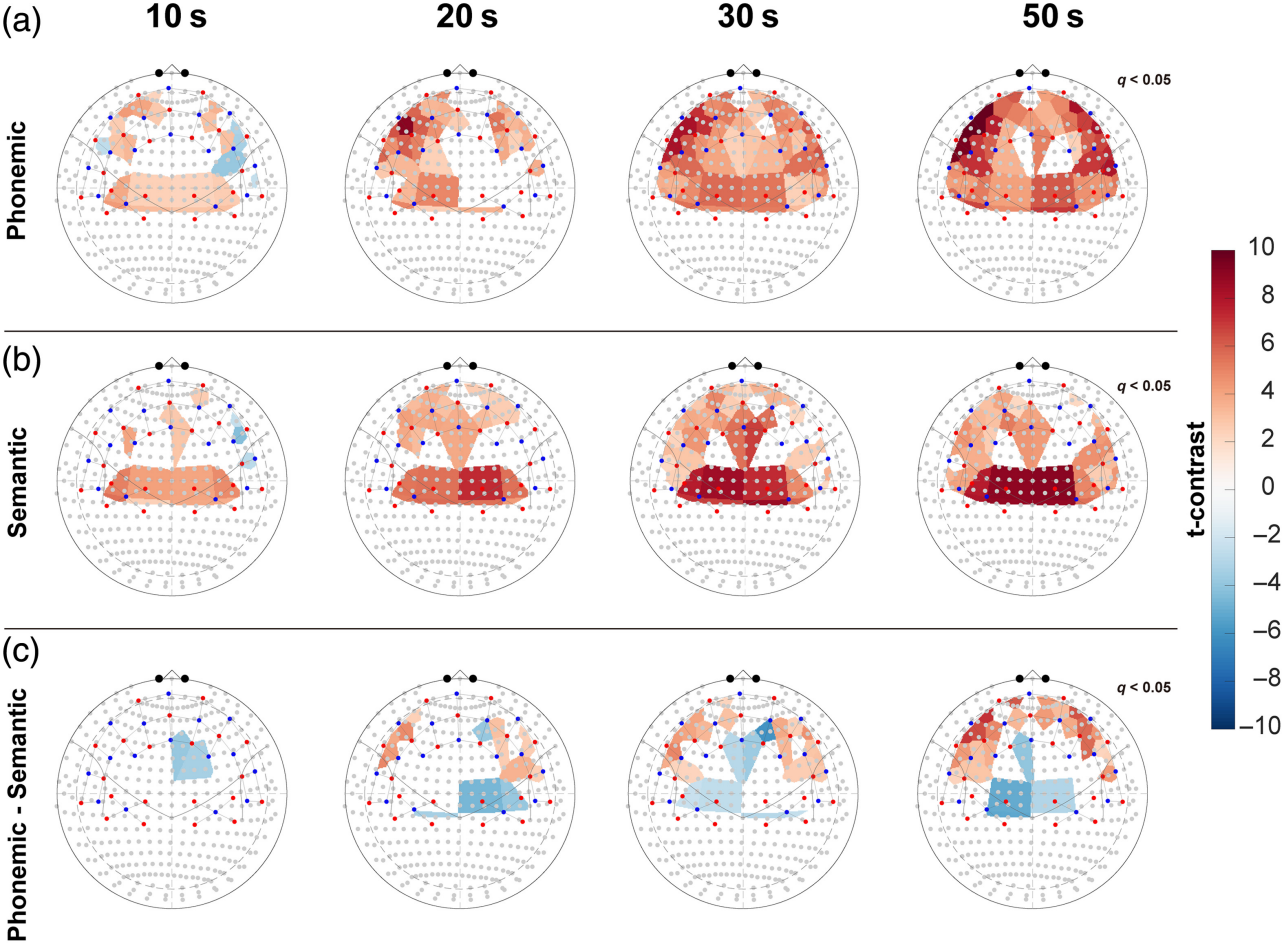
Dynamic HbO responses during pVFT and sVFT, along with the corresponding t-contrasts. Group-level analysis of HbO responses was assessed by varying the length of time considered after the beginning of each task block (stimulus duration): 10, 20, 30, and 50 s. (a) Dynamic HbO responses during pVFT. (b) Dynamic HbO responses during sVFT. (c) Dynamic t-contrast of pVFT and sVFT. At 10 s, significant SMG activation was already observed for both pVFT and sVFT. In the direct contrasts between pVFT and sVFT, stronger activation for pVFT emerged after 20 s across much of the PFC, whereas the SMG showed the opposite pattern. In the sVFT condition, enhanced left-lateralized activation within the PFC became evident after 20 s.

Although the above analyses focused on activation across the whole task block, the following analyses captured the temporal dynamics within shorter time windows. As shown in Fig. 4, significant SMG activation emerged in the first 10 s of both tasks. During this early phase, pVFT additionally recruited five channels in the left BA46 and three in the right BA46 (not yet significant in contrasts), whereas sVFT activation emerged in BA9.

After 10 s, the activation patterns diverged. For pVFT, activity expanded from bilateral BA46 to BA9, IFG, and MTG, with the strongest effects in the left BA46 and IFG; by 30 s, widespread frontal and temporal activation had emerged. For sVFT, activation spread from BA9 to BA46, IFG, and MTG but remained more left-lateralized, with many right BA46/IFG channels showing no significant effects even at 30 s. SMG activation increased for both tasks but increased more rapidly during sVFT.

Direct contrasts showed pVFT > sVFT after 10 s in bilateral IFG and BA46, with both the extent and the t values increasing over time. In comparison, SMG exhibited stronger activation during sVFT, reflected by negative t values.

In summary, SMG activation emerged early in both tasks, but the two VFTs diverged in the onset of frontal activity and in their lateralization patterns. The leftward lateralization observed here, together with the overall finding that pVFT activation was greater than sVFT activation (except in the SMG), was consistent with the analysis of the full-task data.

### 3.4 Task-Based Differences in Functional Connectivity

#### 3.4.1 Channel-level FC differences: pVFT stronger than sVFT, especially in frontotemporal links

**Fig. 5:**
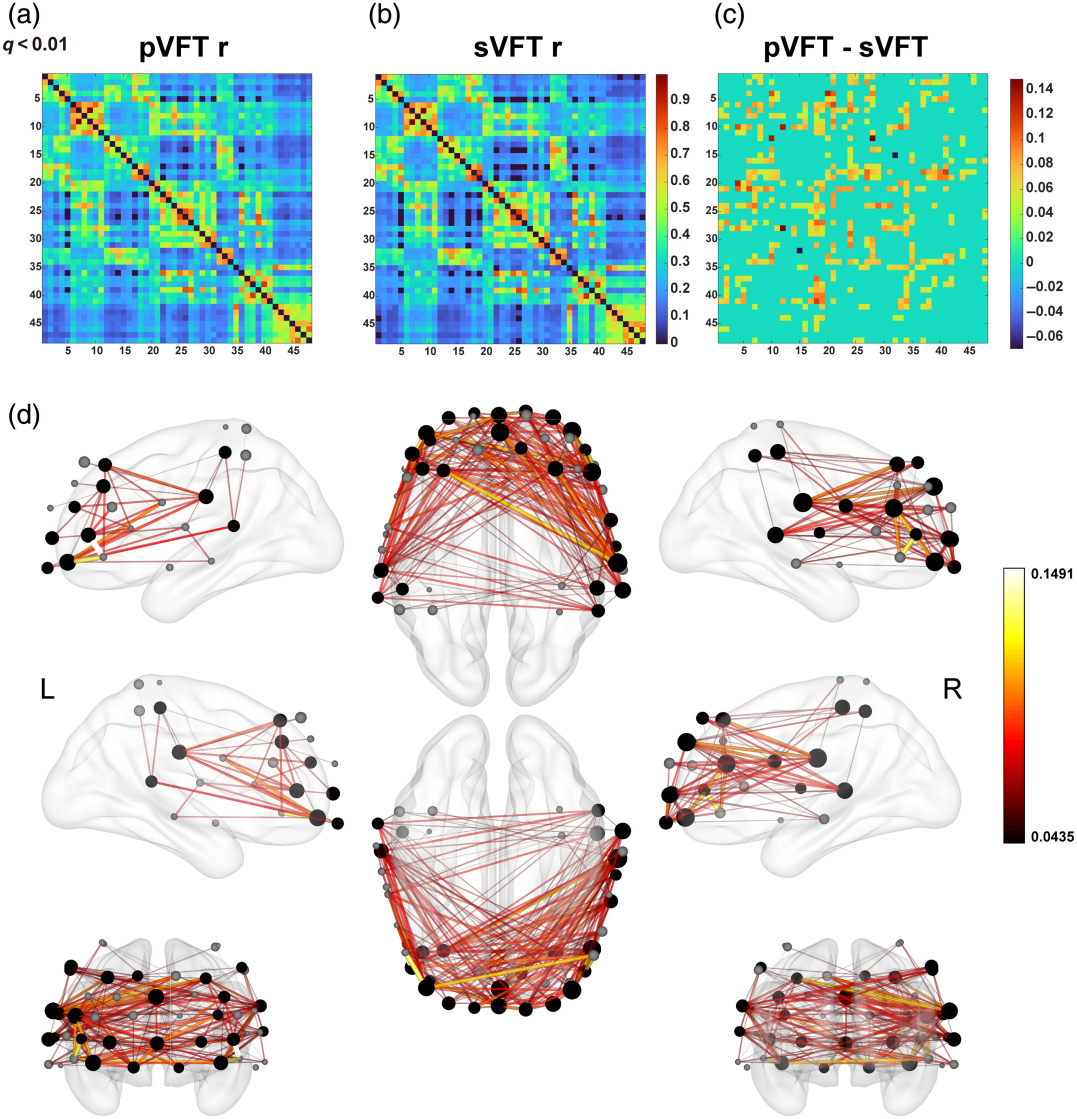
Adjacency matrices and FC contrast maps. (a)–(c) Color-coded group-level Pearson adjacency matrices for pVFT and sVFT, as well as their direct contrast, were computed using the mixed effects connectivity function in the NIRS toolbox. Values with q>0.01 were set to zero. (d) Significant connectivity differences between pVFT and sVFT are projected onto the ICBM152 brain template from multiple viewing angles. Line thickness encodes the magnitude of task-related differences, and line colors follow the colormap shown in the figure. In addition, maps of local weighted node degree differences are displayed on the same brain template. Node-level t-contrasts are represented by sphere size, with gray spheres indicating nonsignificant nodes (p>0.05). Notably, the most significant differences were observed in frontotemporal connections. The lower panel was visualized using BrainNet Viewer.

FC matrices for the group-level pVFT and sVFT conditions, as well as the pVFT vs. sVFT contrast matrix, are shown in Figs. 5(a)–5(c). For all matrices, all values with q>0.01 were set to zero.

For both pVFT and sVFT, the highest Z-values were concentrated in connections between adjacent nodes. Other strong connections were found within ROIs, particularly within the OFC and BA46, including inter-hemispheric connections within these prefrontal regions.

All significant contrasts showed stronger connectivity for pVFT than sVFT. These differences were concentrated in connections between the bilateral temporal and frontal lobes, with the strongest effects observed in right temporal–frontal connections. In addition, a few significant within-IFG connections were identified. Overall, the results indicate that pVFT engages stronger frontotemporal connectivity than sVFT.

For HbR, the group-level FC matrices for pVFT and sVFT showed similar spatial patterns, but the overall connectivity strength was lower than that observed for HbO. Consistent with the HbO results, nearly all significant contrasts indicated stronger connectivity during pVFT than sVFT, but the number of significant connections was smaller. These differences were still primarily concentrated in frontotemporal links, indicating that the stronger frontotemporal connectivity during pVFT was also reflected, albeit more weakly, in the HbR-based FC analysis (Fig. S2 in the Supplementary Material).

#### 3.4.2 Consistent pVFT connectivity advantage across categories confirmed by LME

##### Hemispheric FC: pVFT stronger than sVFT across left, right, and interhemispheric connections

The LME revealed a significant main effect of Condition (β=−0.029, t=−3.69, p<0.001), indicating overall lower connectivity during sVFT. No interaction was found between condition and connection type (p>0.60), suggesting that the task effect was consistent across hemispheric categories.

**Fig. 6:**
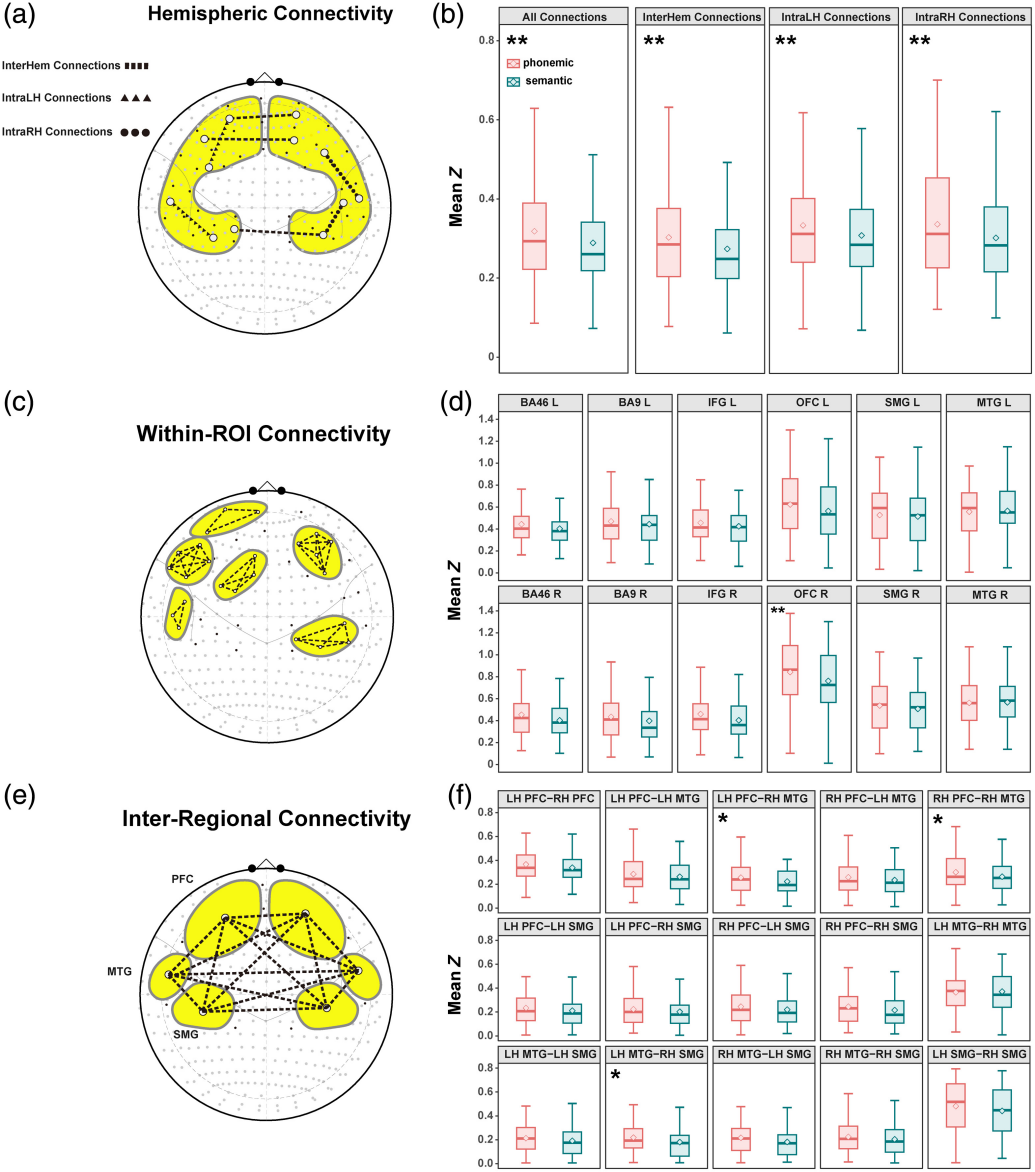
Differences between the two VFTs across connection types. (a), (c), (e) Schematic diagrams illustrating the classification of connection types. (b), (d), (f) Boxplots of mean Z values for pVFT and sVFT within each connection type. For each classification, the significance of the contrasts between tasks was tested using a linear mixed-effects model, with *p<0.05 and **p<0.01 indicating statistical significance. (a), (b) Hemispheric connectivity: intra-left hemisphere, intra-right hemisphere, and inter-hemispheric connections. (c), (d) Within-ROI connectivity: connections confined to each defined ROI. (e), (f) inter-regional connectivity: pairwise connections between the six regions, including the bilateral PFC, MTG, and SMG.

Pairwise tests confirmed that pVFT elicited stronger connectivity than sVFT across all types, including overall (β=0.029, p<0.001), left hemisphere (β=0.026, p=0.0014), right hemisphere (β=0.034, p<0.001), and inter-hemispheric (β=0.029, p<0.001). Within connection types, intra-right hemisphere connectivity was significantly higher than the overall mean (β=0.018, p=0.027). These effects are depicted in Figs. 6(a) and 6(b).

For HbR, the LME similarly revealed a significant main effect of condition (β=−0.016, t=−3.07, p=0.0022), indicating overall lower connectivity during sVFT, with no significant interaction between condition and connection type. Pairwise tests confirmed that pVFT elicited stronger connectivity than sVFT across all connection types, including overall (β=0.016, p=0.002), left hemisphere (β=0.015, p=0.0058), right hemisphere (β=0.020, p<0.001), and inter-hemispheric connections (β=0.016, p=0.0037) [Fig. S3(A) in the Supplementary Material].

##### Within-ROI FC: trend toward pVFT stronger than sVFT except in the MTG

The LME revealed no main effect of condition (β=−0.032, p=0.31) and no condition × connection type interaction. Nonetheless, connectivity strength differed significantly across ROIs irrespective of task: bilateral MTG (LH: β=0.101, p=0.001; RH: β=0.106, p<0.001), OFC (LH: β=0.149, p<0.001; RH: β=0.383, p<0.001), and SMG (LH: β=0.071, p=0.025; RH: β=0.080, p=0.011) all showed stronger connectivity relative to IFG (baseline).

Pairwise contrasts between phonemic and semantic tasks within each regional connection type revealed a general pVFT > sVFT trend across all within-ROI connections except in the MTG. For the pVFT > sVFT contrasts, the highest β values were found in the right IFG (β=0.057) and right OFC (β=0.088). The lowest values occurred in the left SMG (β=0.020) and right SMG (β=0.030); however, only the right OFC connections were statistically significant (p=0.0052).

Interestingly, MTG connections—both left and right—exhibited a reversed pattern, with slightly stronger connectivity during the semantic task, although this trend did not reach statistical significance. These patterns are illustrated in Figs. 6(c) and 6(d).

Descriptively, HbR showed a tendency for higher within-ROI connectivity during pVFT than sVFT across most regions, in line with the HbO results, although none of these differences reached significance. Consistent with HbO, this tendency was not apparent in the MTG [Fig. S3(B) in the Supplementary Material].

##### Inter-ROI FC: HbO showed pVFT stronger than sVFT in R-frontal–R-MTG, L-MTG–R-SMG, L-frontal–R-MTG

The linear mixed-effects model revealed a marginal main effect of condition (β=−0.034, p=0.060), suggesting a trend toward reduced overall connectivity during the semantic fluency task. There was no significant condition × connection type interaction.

Among the 15 inter-ROI connection types, the strongest connectivity was observed in the left SMG–right SMG pair (β=0.169, p<0.001) relative to the baseline left PFC–right PFC pair. By contrast, other connection types showed significantly lower connectivity estimates relative to the baseline (all p<0.001). Pairwise comparisons revealed that connectivity was significantly stronger during phonemic fluency for right frontal–right MTG (β=0.037, p=0.038), left MTG–right SMG (β=0.041, p=0.022), and left frontal–right MTG (β=0.036, p=0.048), consistent with the patterns depicted in Fig. 5(d). Among the 15 inter-ROI connection types, only the left MTG–right MTG connection showed a slight increase in connectivity during the semantic condition relative to phonemic (β=−0.006, p=0.722). These patterns are visualized in Figs. 6(e) and 6(f).

For HbR, the LME model revealed no main effect of condition and no significant condition × connection type interaction. As in the HbO analysis, the strongest inter-ROI connectivity was observed in the left SMG–right SMG pair (β=0.059, p<0.001). Pairwise comparisons showed significantly stronger connectivity during pVFT for the right frontal–right SMG connection (β=0.022, p=0.042). More generally, a descriptive pVFT > sVFT trend was observed across most inter-ROI connections, whereas the left MTG–right MTG connection remained the exception, consistent with the HbO results [Fig. S3(C) in the Supplementary Material].

##### Node degree: higher local and global degrees in pVFT, peaking in BA46/BA9, with further differences in MTG/SMG

**Fig. 7:**
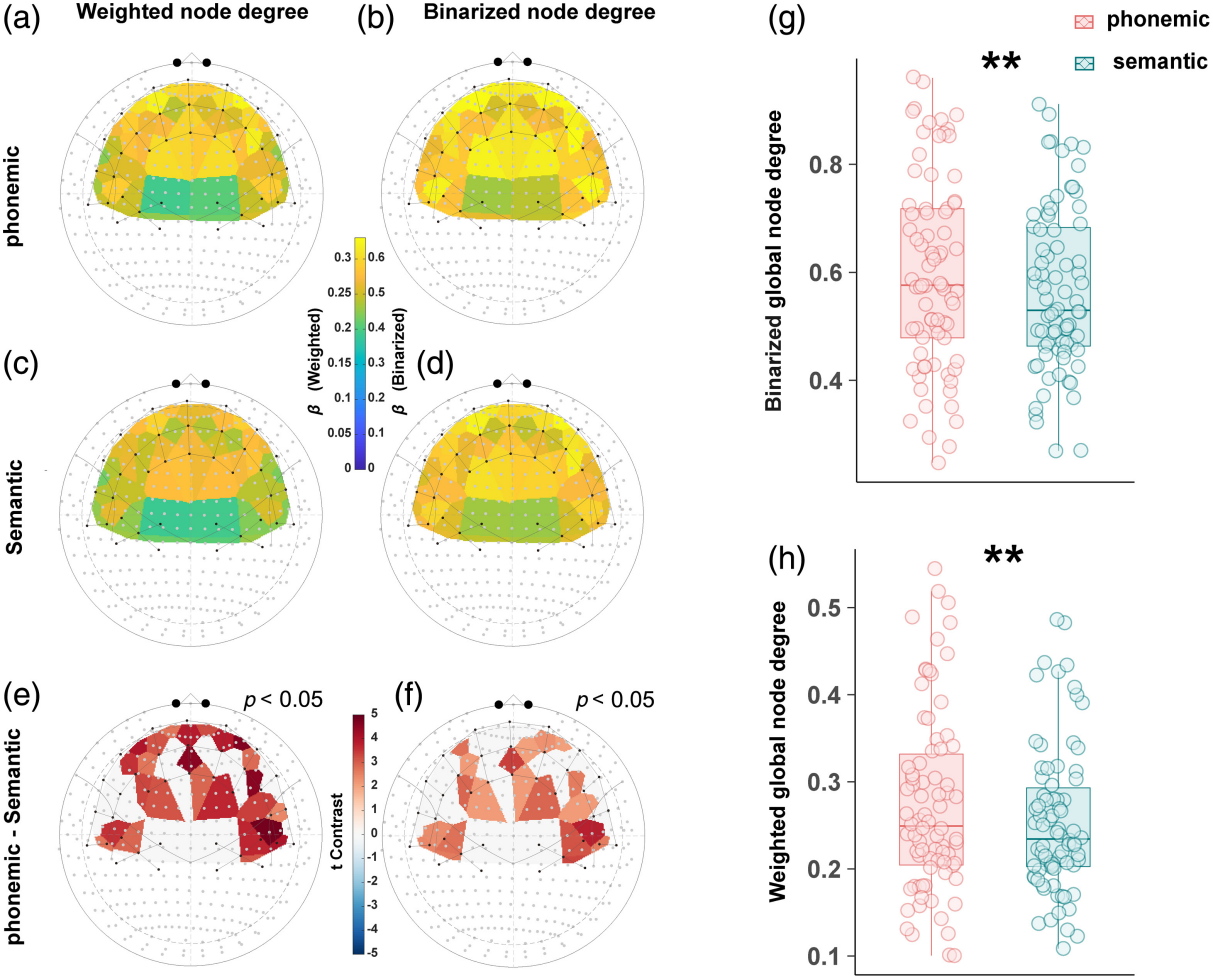
Graph-theoretical differences between pVFT and sVFT tasks. Channel-level beta values of weighted and binarized local node degrees for pVFT (a), (b) and sVFT (c), (d), together with channel-level t-contrast results between the two tasks (e), (f), mapped onto the 10–20 system. All values were derived from linear mixed-effects (LME) models. Beta values and t-contrast values were visualized using separate colorbars; further details are provided in the main text. (g), (h) Boxplots of global node degrees obtained from within-subject t-tests, (g) Binarized global node degree. (h) Weighted global node degree. In both cases, pVFT exhibited significantly higher values than sVFT.

All t-contrasts of weighted local node degrees indicated higher values for pVFT than for sVFT [Fig. 5(d)]. For pVFT, both weighted and binarized local node degrees were highest in the OFC and several channels of BA9 and BA46, with similar spatial patterns across weighted and binarized measures [Figs. 7(a) and 7(b)]. For sVFT, the distribution was comparable but overall lower, as shown in Figs. 7(c) and 7(d).

Figures 7(e) and 7(f) depict significant contrasts (p<0.05). Weighted local node degrees were significantly higher for pVFT in the entire OFC and in several bilateral BA46 and BA9 channels, as well as in parts of the left MTG and SMG. These effects were stronger in the right MTG and SMG than in the left. The binarized node degree pattern largely mirrored the weighted results; although significance in some OFC and BA46 channels was reduced, the effects in BA9, MTG, and SMG persisted.

Differences in both weighted global node degrees (t=3.02, p=0.003) and binarized global node degrees (t=2.87, p=0.005) between pVFT and sVFT were significant [Figs. 7(g) and 7(h)].

For HbR, weighted local node degrees were generally higher during pVFT than sVFT, with peaks in BA9 and BA46 [Figs. S4(A) and S4(C) in the Supplementary Material]. The binarized pattern was similar [Figs. S4(B) and S4(D) in the Supplementary Material], and sVFT showed a broadly comparable but lower distribution. These patterns were consistent with the HbO results, although OFC involvement was less prominent and significant contrast effects were more spatially restricted. Specifically, no significant differences were observed in the OFC or in several channels of the left BA46, left MTG, and left SMG [Figs. S4(E) and S4(F) in the Supplementary Material]. Likewise, although both weighted and binarized global node degrees were numerically higher for pVFT, neither reached significance (weighted: t=1.69, p=0.095; binarized: t=1.38, p=0.171), consistent with the overall weaker HbR topology pattern [Figs. S4(G) and S4(H) in the Supplementary Material].

### 3.5 Lack of individual behavioral and executive function influence on neural patterns

**Fig. 8:**
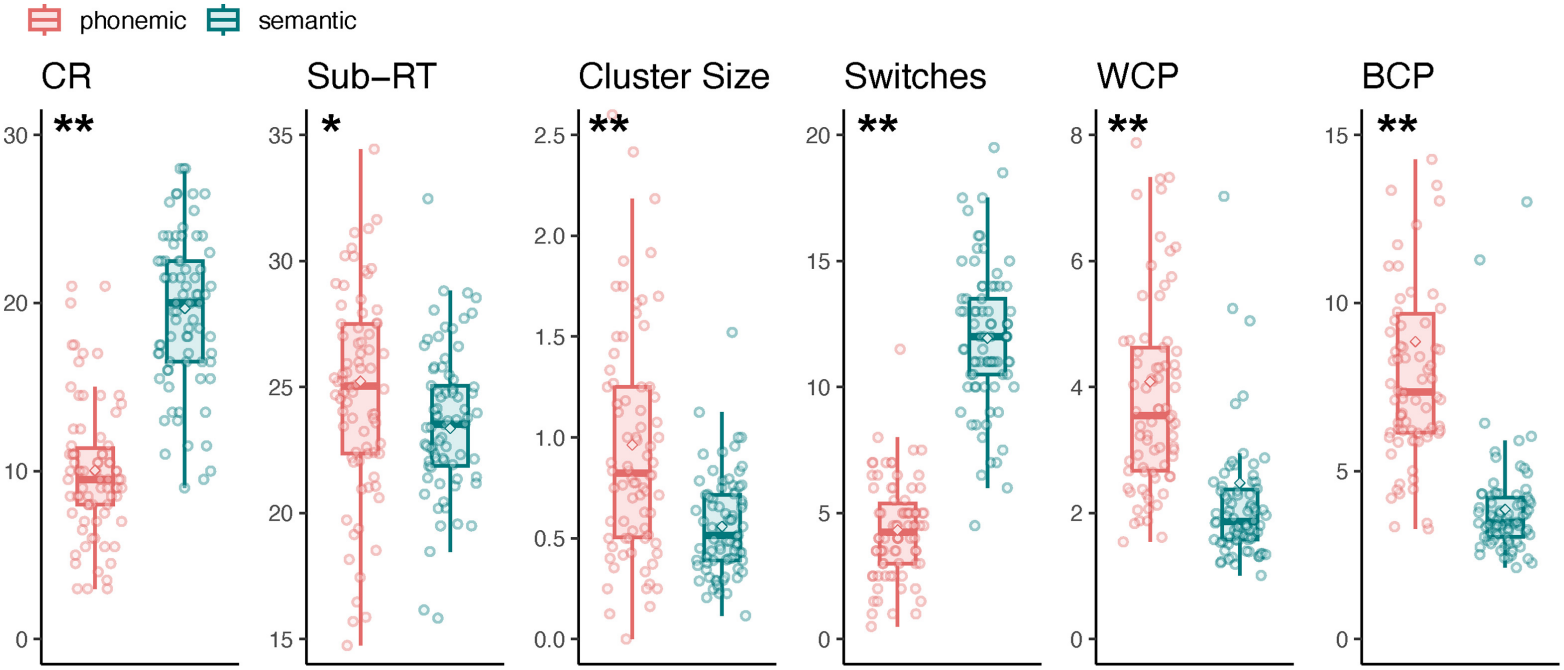
Participants’ behavioral variables in pVFT and sVFT. Boxplots with overlaid dot plots of participants’ behavioral variables in pVFT and sVFT. For both conditions, values represent the average across the two task blocks.

After FDR correction, paired-samples t-tests revealed significant differences between the two VFTs across all behavioral variables, including CR (t=−21.6, p<0.001), switches (t=−25.9, p<0.001), BCP (t=14.6, p<0.001), WCP (t=10.3, p<0.001), average cluster size (t=5.88, p<0.001), and sub-RT (t=2.34, p=0.022). These differences are illustrated in Fig. 8.

Except for a significant main effect of the cluster size in the right IFG and a significant main effect of BCP in the right SMG, no other variables showed significant main or interaction effects in the LME analyses, as shown in Table 2. This indicates that almost all VFT behavioral variables were not significantly associated with VFT activation levels, and task type did not moderate the relationship between behavioral performance and activation. Furthermore, difference correlation analyses between the pVFT–sVFT behavioral score differences and the corresponding activation differences in each brain region revealed no significant behavioral–brain region pairs, as reported in Data S3 in the Supplementary Material. Consistent with the HbO results, no significant effects were observed in the corresponding HbR analyses, either in the LME models or in the difference correlation analyses (Data S4 and S5 in the Supplementary Material).

**Table 2:** p-Values for the main effects of behavioral variables and their interaction with condition on activation levels across ROIs.

	BA46_L	BA46_R	BA9_L	BA9_R	IFG_L	IFG_R	MTG_L	MTG_R	OFC_L	OFC_R	SMG_L	SMG_R
BCP	0.695	0.245	0.297	0.059	0.852	0.698	0.656	0.734	0.769	0.425	0.076	**0.032**
BCP × task	0.592	0.252	0.894	0.406	0.525	0.26	0.357	0.081	0.762	0.69	0.912	0.578
CR	0.749	0.836	0.407	0.568	0.67	0.638	0.672	0.607	0.987	0.545	0.542	0.467
CR × task	0.696	0.636	0.396	0.29	0.853	0.702	0.348	0.539	0.415	0.14	0.723	0.956
Cluster size	0.346	0.097	0.344	0.093	0.263	**0.019**	0.266	0.21	0.664	0.834	0.731	0.426
Cluster size × task	0.372	0.403	0.708	0.59	0.664	0.241	0.603	0.198	0.398	0.275	0.925	0.549
Sub-RT	0.452	0.353	0.215	0.185	0.495	0.734	0.322	0.47	0.802	0.871	0.438	0.439
Sub-RT × task	0.576	0.226	0.431	0.249	0.884	0.683	0.968	0.845	0.295	0.196	0.343	0.268
Switches	0.467	0.31	0.18	0.05	0.392	0.351	0.981	0.2	0.798	0.311	0.197	0.112
Switches × task	0.98	0.508	0.371	0.124	0.951	0.865	0.36	0.497	0.654	0.165	0.364	0.685
WCP	0.31	0.682	0.072	0.186	0.492	0.715	0.567	0.983	0.157	0.213	0.156	0.059
WCP × task	0.683	0.586	0.178	0.854	0.902	0.206	0.82	0.245	0.44	0.614	0.684	0.54

All p-values are uncorrected for multiple comparisons. Bold values indicate uncorrected p<0.05. A significant main effect of the cluster size was observed in the right IFG and a significant main effect of BCP in the right SMG; however, neither effect survived FDR correction.

None of the executive function variables showed significant associations with VFT activation levels, and the task effects were not dependent on the level of executive function, as shown in Table 3. Consistent with the HbO results, no significant associations were observed in the corresponding HbR analyses (Data S6 in the Supplementary Material). All three executive function tasks showed robust condition effects on both reaction time and accuracy: RTs increased with task difficulty, whereas accuracy decreased, as reported in Sec. 1 in the Supplementary Material. This demonstrates that the difficulty manipulations were effective and reliably engaged cognitive control processes, confirming the sensitivity of these tasks for assessing executive functions.

**Table 3:** p-Values for the main effects of executive function test scores and their interaction with condition on activation levels across ROIs.

	BA46_L	BA46_R	BA9_L	BA9_R	IFG_L	IFG_R	MTG_L	MTG_R	OFC_L	OFC_R	SMG_L	SMG_R
2back accuracy	0.926	0.981	0.989	0.757	0.685	0.505	0.706	0.319	0.413	0.546	0.777	0.579
2back accuracy × task	0.958	0.855	0.906	0.72	0.83	0.659	0.925	0.41	0.596	0.816	0.73	0.996
Shift difference	0.795	0.866	0.825	0.888	0.828	0.669	0.214	0.139	0.507	0.443	0.853	0.591
Shift difference × task	0.75	0.786	0.96	0.647	0.851	0.831	0.484	0.921	0.313	0.684	0.506	0.396
Stroop ratio	0.769	0.166	0.722	0.136	0.826	0.118	0.724	0.139	0.491	0.818	0.155	0.888
Stroop ratio × task	0.105	0.388	0.103	0.652	0.405	0.336	0.833	0.424	0.46	0.589	0.074	0.668

All p-values are uncorrected for multiple comparisons, and no significant effects were observed. The specific measures used were the Stroop ratio for inhibition, 2-back accuracy for updating, and the shifting cost (shift difference) for shifting.

## 4 Discussion

### 4.1 Main Findings

Our results highlight a robust and stable neural dissociation between phonemic and semantic fluency. We report three main findings that delineate nonoverlapping neurocognitive systems for phonemic (pVFT) and semantic (sVFT) fluency. Double dissociation of regional activation: pVFT robustly engaged the PFC, including bilateral IFG, DLPFC, and OFC, whereas sVFT more strongly engaged bilateral SMG, with stronger leftward lateralization for sVFT in IFG/SMG. Distinct network states: pVFT induced higher global and local integration and strengthened long-range PFC–temporal/parietal connectivity. Behavior–neural dissociation: despite greater verbal output during sVFT, pVFT elicited stronger PFC activation, and these neural differences were not explained by in-task performance or general executive measures.

Together, these findings challenge the practice of treating pVFT and sVFT as interchangeable in neuroimaging.

### 4.2 Stable Behavioral–Neural Dissociation Between pVFT and sVFT

Our findings reveal a striking behavioral–neural dissociation between pVFT and sVFT. Behaviorally, sVFT yielded more correct responses and switches, consistent with prior studies.^1^^,^^32^^,^^53^ Yet, paradoxically, the lower-output pVFT elicited significantly stronger and more widespread activation in the PFC. This contrast highlights that the key distinction lies not in the quantity of output but in the cognitive cost per unit of output. Guided by established semantic networks, sVFT retrieval and switching follow low-cost associative pathways.^54^^,^^55^ By contrast, pVFT lacks intrinsic structure, requiring repeated high-cost resets, longer pauses, and strategic re-initiations. Each retrieval, therefore, demands intensive DLPFC control, left IFG selection and inhibition, and OFC monitoring.

Importantly, these neural differences were not modulated by individual task performance or general executive ability assessed in this study. We found no significant main or interaction effects of behavioral scores or executive function measures on ROI activation. This rules out “difficulty,” “performance,” and “executive function” hypotheses: A difficulty hypothesis would predict that participants who found pVFT harder (a larger performance gap compared with sVFT) should show greater PFC activation differences, but our difference correlation analyses did not support this. If the dissociation were driven by general executive function, individuals with higher capacity (e.g., lower Stroop ratios) should exhibit different neural patterns, which was not observed. A performance hypothesis would expect stronger SMG activation in sVFT to correlate with word output, yet no such relationship emerged. The absence of these correlations suggests that the sustained SMG activation in sVFT reflects a stable, task-inherent property of high-throughput processing rather than a linear function of individual performance.

Collectively, these findings support our conclusion that the observed neural dissociation reflects a fundamental difference in the cognitive architecture and neural systems required for each task. The brain employs different types of processing for each VFT variant: pVFT imposes a high-cost executive control burden, whereas sVFT leverages high-throughput semantic pathways. This represents a stable, task-inherent property, not a product of individual ability or effort. This task-driven dissociation, rather than performance-driven variability, represents a core contribution of the present study.

### 4.3 Distinct Cortical Activation Patterns

#### 4.3.1 Stronger prefrontal engagement in pVFT

Our results demonstrated that pVFT activation was consistently greater than sVFT activation in nearly all PFC regions, particularly in the bilateral IFG, DLPFC, and OFC. This finding is consistent with prior evidence indicating that the PFC plays a crucial role in pVFT.^27^^–^^29^^,^^56^ The stronger PFC engagement likely reflects the greater executive demands of pVFT. Unlike the semantically guided and relatively automated retrieval in sVFT,^54^ pVFT requires a constrained, strategy-dependent search,^57^^,^^58^ which in turn relies on the coordinated contributions of multiple PFC subregions. The DLPFC is central for initiating and sustaining a strategic search plan, manipulating working memory, and monitoring output.^59^ It also supports action initiation in phonological searches.^60^ Because phonemic rules are arbitrary (e.g., words beginning with /d/), the lexical space lacks intrinsic structure and thus requires constant top-down guidance. Stuss et al.^29^ found the DLPFC most affected by pVFT, and our dynamics further revealed earlier BA46 engagement, suggesting task-set establishment for high-control, unpracticed tasks. The left IFG contributes to cognitive control by resolving lexical competition and selecting appropriate items from activated candidates.^61^ In pVFT, participants must actively suppress dominant semantic associations to follow phonemic rules, a process that heavily taxes left IFG resources.^62^ The OFC showed the largest task-related difference. Prior fNIRS studies have reported OFC involvement in verbal tasks.^63^ Its role in behavioral monitoring, impulse control, and inhibition,^64^ and in evaluating/switching among competing goals,^65^ underscores its critical role in pVFT. The need to continuously monitor output and avoid rule violations likely explains the markedly stronger OFC activation.

#### 4.3.2 Comparable MTG involvement in both tasks

We found no significant difference in activation in the middle temporal gyrus (MTG), a core region for lexical–semantic processing. Although sVFT is thought to rely heavily on the temporal lobe,^66^ its MTG activation was not greater than that observed during pVFT. A likely explanation is that pVFT, despite phonemic constraints, still searches broadly within the lexical–semantic network, leading both tasks to engage the temporal system comparably. Consistent with prior reports, pVFT also involves temporal-lobe mechanisms.^32^^,^^67^ Thus, the primary source of dissociation lies in the PFC, reflecting the high-cost executive control required for pVFT rather than differences in semantic system engagement.

#### 4.3.3 Role of the supramarginal gyrus in sVFT

Bilateral SMG activation was robust in both tasks, consistent with prior findings that link the parietal cortex, particularly the SMG, to fluency performance.^28^^,^^29^^,^^44^ However, our results suggest a stronger association with sVFT, consistent with findings that link SMG structure to semantic fluency performance,^68^ despite lesion studies highlighting its role in pVFT.^32^ This challenges the classic view of the SMG as primarily phonological and instead supports the view of a more integrative role in language production, where enhanced SMG activity during sVFT relates to successful word output rather than phonological rules.^69^

Several mechanisms may underlie this effect. The SMG supports the phonological store of working memory, which is refreshed more frequently under the high-output demands of sVFT.^45^^,^^69^^–^^71^ It also mediates semantic-to-phonological mapping by converting abundant semantic candidates into articulable forms.^72^^–^^74^ In addition, auditory–motor integration through dorsal-stream processes ensures fluent articulation.^75^^,^^76^

Dynamic analysis revealed early SMG activation in both tasks, reflecting its general role in speech preparation, but only sVFT showed sustained and stronger activation, consistent with continuous high-throughput output. By contrast, pVFT’s bottleneck lies in frontal strategic search, resulting in reduced SMG involvement. Thus, the enhanced SMG role in sVFT reflects its capacity to support efficient lexical throughput, rather than phonological processing alone.

#### 4.3.4 Task-related differences in hemispheric lateralization

This study corroborates the well-established left-hemisphere dominance in frontal activation during VFTs, consistent with lesion and neuroimaging evidence. Importantly, we observed that sVFT showed stronger leftward lateralization than pVFT in the IFG and SMG. The heavier control demands of pVFT may require broader, bilateral recruitment of right-hemisphere homologs to support the effortful search process, thereby weakening lateralization. This aligns with reports that the right PFC can be recruited under high cognitive load,^77^ although its contribution to verbal fluency remains limited.^78^

Beyond the conventional interpretation of leftward dominance as reflecting language-related frontal specialization, the present finding may also be contextualized within the approach-withdrawal motivation framework, in which relatively greater left prefrontal activity has been linked to approach-related engagement and active goal pursuit.^79^^,^^80^ In this sense, the leftward lateralization observed here may also be related to the sustained verbal search and task engagement required during VFT performance. However, this interpretation is necessarily tentative. Hemispheric asymmetry in the PFC may also be modulated by environmental and situational factors, such as body posture, testing conditions, individual trait differences, circadian timing, or other state-related influences.^81^^,^^82^ As these variables were not directly assessed in the current study, their contribution to the observed lateralization patterns cannot be ruled out.

### 4.4 Task-Related Functional Network Reorganization

Beyond regional dissociations, FC analyses demonstrated that pVFT consistently elicited stronger and more widespread connectivity than sVFT. Graph-theoretical measures revealed significantly higher global node degrees (weighted and binarized) and elevated local node degrees, especially in the PFC, right MTG, and right SMG. This suggests that pVFT demands a more integrated network state, requiring continuous communication across regions to sustain a complex task set, search for nonobvious exemplars, and monitor performance.

The most substantial differences (pVFT > sVFT) appeared in long-range connections linking the PFC with bilateral temporal lobes and SMG. This aligns with disconnectome-based evidence from large-scale VFT lesion-symptom mapping^78^ and offers a network-level explanation for our activation patterns, indicating that pVFT relies more on frontal–posterior interplay, consistent with models of top-down executive control over lexical and phonological representations.

Within-ROI connectivity also diverged. Prefrontal regions exhibited stronger within-ROI coupling in pVFT, whereas bilateral MTG—central to semantic processing—showed a slight, nonsignificant trend toward higher connectivity in sVFT. This pattern suggests purposeful reweighting rather than global upregulation. During pVFT, the PFC acts as a command hub, boosting its communication while reducing MTG centrality; sVFT instead relies more economically on temporal-lobe semantic pathways, with the PFC serving primarily a supervisory role.

Overall, pVFT requires high-cost executive control, reflected not only in stronger PFC activation but also in a PFC-led network state of enhanced global integration. By contrast, sVFT leverages existing semantic pathways in a more efficient, modular organization. This network reorganization may represent the neural cost of executing a strategy-driven, nonautomated language task.

### 4.5 Methodological Implications for Neuroimaging Research

This study offers important methodological insights. We show that the neural dissociation between pVFT and sVFT is a stable, task-driven phenomenon, independent of individual performance or executive ability. This underscores the reliability and robustness of the VFT paradigm when its two variants are clearly distinguished. This directly addresses a key motivation of this study: in fNIRS research, pVFT and sVFT are often used interchangeably or without clear justification.^20^ Our findings provide empirical evidence that such practice is untenable. Because the two tasks probe different neurocognitive systems, their careful selection is critical.

Based on our activation and network results, we recommend three methodological considerations for future studies. First, task selection should be guided by the target circuitry: pVFT robustly engages the bilateral PFC (including IFG, DLPFC, and OFC) and elicits a high-integration state, whereas sVFT emphasizes SMG-centered circuits and a more modular, temporal-lobe-weighted state. Second, researchers should avoid cross-generalization, as inferences drawn from one variant cannot be directly transferred to the other without explicit testing. Third, the dissociation between pVFT and sVFT should itself be leveraged as an informative contrast, rather than relying solely on absolute activation within a single task.

### 4.6 Clinical Implications

Because the dissociation between pVFT and sVFT is a stable, task-driven phenomenon, which demonstrates the reliability and robustness of the VFT paradigm, these findings can be translated into several important clinical implications:

Divergent profiles, such as impaired pVFT with preserved sVFT or vice versa, can localize dysfunction to frontal executive circuits versus semantic–phonological interfacing, thereby enhancing diagnostic specificity. This pattern-level diagnosis may be more precise than relying on single-task activation, for example, using phonemic fluency to detect frontal damage,^53^ and can help distinguish specific circuit dysfunction from generalized impairment.

Rehabilitation targeting. Because pVFT and sVFT engage distinct networks, their dissociation can inform both targeted therapy and recovery monitoring. pVFT-related deficits in regions such as the DLPFC, IFG, and OFC may be addressed through training of strategic control and inhibition, whereas sVFT-related deficits in the SMG and semantic–phonological mapping call for therapies that enhance semantic activation and articulation. Repeated use of these tasks during rehabilitation also enables clinicians to track network-specific recovery, with improvements in pVFT reflecting restored frontal control and improvements in sVFT reflecting recovery of semantic pathways.

Assessment design. The dissociation between pVFT and sVFT offers a tool for differentiating network-specific dysfunctions in patients. pVFT primarily probes frontal executive circuits (IFG/DLPFC/OFC), whereas sVFT targets SMG-centered semantic pathways. Comparing their activation and connectivity profiles helps clinicians distinguish frontal control deficits from semantic–phonological impairments, thereby improving diagnostic specificity and guiding more precise clinical imaging assessments.

Pattern-based biomarkers. Because the neural signatures of pVFT and sVFT are stable and distinct, the dissociation pattern between the two tasks may surpass either task alone in sensitivity for early detection, patient profiling, and treatment monitoring.

### 4.7 Limitations and Future Considerations

fNIRS offers limited spatial resolution and does not capture subcortical activity, but these constraints primarily affect fine-grained circuit inference rather than the cortical-level contrasts emphasized here. Multimodal combinations (e.g., EEG–fNIRS) could further enrich spatiotemporal mapping in future studies.

Generalizability beyond healthy young adults remains to be established in clinical and lifespan samples, ideally via head-to-head task comparisons to validate pattern-based biomarkers.

The present study employed a phonemic task based on the Chinese Pinyin system. Although we consider this a “pure” phonemic task that can effectively probe phonological retrieval mechanisms, the extent to which the results can be generalized to alphabet-based languages remains to be determined and should be tested in future cross-linguistic studies.

## 5 Conclusion

This study demonstrates that phonemic and semantic fluency tasks possess distinct and stable neural signatures: pVFT predominantly engages a PFC-driven executive network with high global integration, whereas sVFT depends more on an SMG-centered circuit for efficient lexical output. These differences are task-intrinsic, not modulated by individual behavior or executive function. Our findings address methodological ambiguity in fNIRS-VFT research and provide a framework for precision neuroscience and clinical assessment—emphasizing that task selection must be guided by targeted neural circuitry.

## Supplementary Material

10.1117/1.NPh.13.2.025009.s01

## Data Availability

The code and data would be made available upon request to the authors after a formal data-sharing agreement has been reached.

## References

[1] Bose A. et al., “Verbal fluency difficulties in aphasia: a combination of lexical and executive control deficits,” Int. J. Lang. Commun. Disord. 57(3), 593–614 (2022). 10.1111/1460-6984.12710 35318784 PMC9314833

[r2] Troyer A. K. Moscovitch M. Winocur G., “Clustering and switching as two components of verbal fluency: evidence from younger and older healthy adults,” Neuropsychology 11(1), 138 (1997). NEUPEG 10.1037/0894-4105.11.1.138 9055277

[r3] Henry J. D. Crawford J. R., “A meta-analytic review of verbal fluency performance following focal cortical lesions,” Neuropsychology 18(2), 284–295 (2004). NEUPEG 10.1037/0894-4105.18.2.284 15099151

[4] Lezak M. D., Neuropsychological assessment, 3rd ed., Oxford University Press (1995).

[5] Ruff R. M. et al., “The psychological construct of word fluency,” Brain Lang. 57(3), 394–405 (1997). 10.1006/brln.1997.1755 9126423

[6] Kraan C. Stolwyk R. J. Testa R., “The abilities associated with verbal fluency performance in a young, healthy population are multifactorial and differ across fluency variants,” Appl. Neuropsychol. Adult 20(3), 159–168 (2013). 10.1080/09084282.2012.670157 23383872

[7] Bittner R. M. Crowe S. F., “The relationship between working memory, processing speed, verbal comprehension, and FAS performance following traumatic brain injury,” Brain Inj. 21(7), 709–719 (2007). BRAIEO 1362-301X 10.1080/02699050701468917 17653945

[r8] Patra A. Bose A. Marinis T., “Lexical and cognitive underpinnings of verbal fluency: evidence from Bengali-English bilingual aphasia,” Behav. Sci. 10(10), 155 (2020). 10.3390/bs10100155 33050055 PMC7600573

[r9] Shao Z. et al., “What do verbal fluency tasks measure? Predictors of verbal fluency performance in older adults,” Front. Psychol. 5, 772 (2014).1664-1078 10.3389/fpsyg.2014.00772 25101034 PMC4106453

[10] Troyer A. K., “Normative data for clustering and switching on verbal fluency tasks,” J. Clin. Exp. Neuropsychol. 22(3), 370–378 (2000). JCENE8 10.1076/1380-3395(200006)22:3;1-V;FT370 10855044

[11] Fisk J. E. Sharp C. A., “Age-related impairment in executive functioning: updating, inhibition, shifting, and access,” J. Clin. Exp. Neuropsychol. 26(7), 874–890 (2004). JCENE8 10.1080/13803390490510680 15742539

[r12] Unsworth N. Spillers G. J. Brewer G. A., “Variation in verbal fluency: a latent variable analysis of clustering, switching, and overall performance,” Q. J. Exp. Psychol. 64(3), 447–466 (2011). QJXPAR 0033-555X 10.1080/17470218.2010.505292 20839136

[r13] Abrahams S. et al., “Verbal fluency and executive dysfunction in amyotrophic lateral sclerosis (ALS),” Neuropsychologia 38(6), 734–747 (2000). NUPSA6 0028-3932 10.1016/S0028-3932(99)00146-3 10689049

[14] Piatt A. L. et al., “Action (verb naming) fluency as an executive function measure: convergent and divergent evidence of validity,” Neuropsychologia 37(13), 1499–1503 (1999). NUPSA6 0028-3932 10.1016/S0028-3932(99)00066-4 10617270

[15] Amunts J. et al., “Executive functions predict verbal fluency scores in healthy participants,” Sci. Rep. 10(1), 11141 (2020). SRCEC3 2045-2322 10.1038/s41598-020-65525-9 32636406 PMC7341845

[16] Husain S. F. et al., “Functional near-infrared spectroscopy of English-speaking adults with attention-deficit/hyperactivity disorder during a verbal fluency task,” J. Atten. Disord. 27(13), 1448–1459 (2023). 10.1177/10870547231180111 37341192

[r17] Itakura M. et al., “Association between social functioning and prefrontal cortex function during a verbal fluency task in schizophrenia: a near-infrared spectroscopic study,” Psychiatry Clin. Neurosci. 71(11), 769–779 (2017). 10.1111/pcn.12548 28657683

[r18] Ghanavati E. et al., “Differential role of prefrontal, temporal and parietal cortices in verbal and figural fluency: implications for the supramodal contribution of executive functions,” Sci. Rep. 9(1), 3700 (2019). SRCEC3 2045-2322 10.1038/s41598-019-40273-7 30842493 PMC6403289

[r19] Tassi E. et al., “A scoping review of near infrared spectroscopy studies employing a verbal fluency task in bipolar disorder,” J. Affect. Disord. 298(Pt A), 604–617 (2022). JADID7 10.1016/j.jad.2021.11.019 34780861

[20] Ren Y. et al., “A scoping review of utilization of the verbal fluency task in Chinese and Japanese clinical settings with near-infrared spectroscopy,” Front. Psychiatry 15, 1282546 (2024). 10.3389/fpsyt.2024.1282546 38525251 PMC10957746

[21] Boas D. A. et al., “Twenty years of functional near-infrared spectroscopy: introduction for the special issue,” NeuroImage 85(Pt 1), 1–5 (2014). NEIMEF 1053-8119 10.1016/j.neuroimage.2013.11.033 24321364

[22] Butler L. K. Kiran S. Tager-Flusberg H., “Functional near-infrared spectroscopy in the study of speech and language impairment across the life span: a systematic review,” Am. J. Speech Lang. Pathol. 29(3), 1674–1701 (2020). 10.1044/2020_AJSLP-19-00050 32640168 PMC7893520

[23] Luo L. Luk G. Bialystok E., “Effect of language proficiency and executive control on verbal fluency performance in bilinguals,” Cognition 114(1), 29–41 (2010). CGTNAU 0010-0277 10.1016/j.cognition.2009.08.014 19793584

[r24] Paap K. R. et al., “No compelling evidence for a bilingual advantage in switching or that frequent language switching reduces switch cost,” J. Cogn. Psychol. 29(2), 89–112 (2017). 10.1080/20445911.2016.1248436

[r25] Sandoval T. C. et al., “What causes the bilingual disadvantage in verbal fluency? The dual-task analogy,” Biling. Lang. Cogn. 13(2), 231–252 (2010). 10.1017/S1366728909990514

[26] Thiele K. Quinting J. M. Stenneken P., “New ways to analyze word generation performance in brain injury: a systematic review and meta-analysis of additional performance measures,” J. Clin. Exp. Neuropsychol. 38(7), 764–781 (2016). JCENE8 10.1080/13803395.2016.1163327 27171352

[27] Baldo J. V. Shimamura A. P., “Letter and category fluency in patients with frontal lobe lesions,” Neuropsychology 12(2), 259–267 (1998). NEUPEG 10.1037/0894-4105.12.2.259 9556772

[r28] Baldo J. V. et al., “Role of frontal versus temporal cortex in verbal fluency as revealed by voxel-based lesion symptom mapping,” J. Int. Neuropsychol. Soc. 12(6), 896–900 (2006). JCENE8 10.1017/S1355617706061078 17064451

[29] Stuss D. T. et al., “The effects of focal anterior and posterior brain lesions on verbal fluency,” J. Int. Neuropsychol. Soc. 4(3), 265–278 (1998). JCENE8 10.1017/S1355617798002653 9623001

[30] Duan C. et al., “An fNIRS-based investigation of cerebral hemodynamic responses during verbal fluency task and n-back task in individuals with mild cognitive impairment,” Front. Neurol. 16, 1571964 (2025). 10.3389/fneur.2025.1571964 40406705 PMC12094942

[31] Biesbroek J. M. et al., “Shared and distinct anatomical correlates of semantic and phonemic fluency revealed by lesion-symptom mapping in patients with ischemic stroke,” Brain Struct. Funct. 221(4), 2123–2134 (2016). 10.1007/s00429-015-1033-8 25939335 PMC4853441

[32] Chouiter L. et al., “Partly segregated cortico-subcortical pathways support phonologic and semantic verbal fluency: a lesion study,” Neuroscience 329, 275–283 (2016). 10.1016/j.neuroscience.2016.05.029 27217213

[33] Peng Z. et al., “The role of the bilateral inferior frontal gyrus in non-suicidal self-injury (NSSI) among depressed adolescents: a functional near-infrared spectroscopy (fNIRS) study during verbal fluency tasks,” J. Psychiatr. Res. 180, 418–427 (2024). JPYRA3 0022-3956 10.1016/j.jpsychires.2024.11.005 39536503

[34] Zhang Y. et al., “Prefrontal brain activity and self-injurious behavior in adolescents with major depressive disorder: a functional near-infrared spectroscopy (fNIRS) study,” J. Psychiatr. Res. 176, 248–253 (2024). JPYRA3 0022-3956 10.1016/j.jpsychires.2024.06.001 38897055

[35] Li J. et al., “Brain activation characteristics of schizophrenia and bipolar disorder-I patients during letter and category fluency tasks: an empirical study using functional near-infrared spectroscopy,” J. Affect. Disord. 382, 471–477 (2025). JADID7 10.1016/j.jad.2025.04.073 40274115

[36] Wang K. et al., “Chinese verbal fluency deficiency in temporal lobe epilepsy with and without hippocampal sclerosis: a multi-scale study,” J. Neurosci. 44(37), e0558242024 (2024). JNRSDS 0270-6474 10.1523/JNEUROSCI.0558-24.2024 39054070 PMC11391496

[37] Boersma P. Weenink D., “Praat: doing phonetics by computer [Computer program],” Version 6.4.42, https://praat.org (Retrieved 28 August 2025).

[38] Miyake A. et al., “The unity and diversity of executive functions and their contributions to complex ‘Frontal Lobe’ tasks: a latent variable analysis,” Cogn. Psychol. 41(1), 49–100 (2000). CGPSBQ 0010-0285 10.1006/cogp.1999.0734 10945922

[39] Salthouse T. A. Atkinson T. M. Berish D. E., “Executive functioning as a potential mediator of age-related cognitive decline in normal adults,” J. Exp. Psychol. Gen. 132(4), 566–594 (2003). JPGEDD 1939-2222 10.1037/0096-3445.132.4.566 14640849

[40] Owen A. M. et al., “N-back working memory paradigm: a meta-analysis of normative functional neuroimaging studies,” Hum. Brain Mapp. 25(1), 46–59 (2005). HBRME7 1065-9471 10.1002/hbm.20131 15846822 PMC6871745

[41] Stroop J. R., “Studies of interference in serial verbal reactions,” J. Exp. Psychol. 18, 643–662 (1935). JEPSAK 0022-1015 10.1037/h0054651

[42] Kono T. et al., “Multiple-time replicability of near-infrared spectroscopy recording during prefrontal activation task in healthy men,” Neurosci. Res. 57(4), 504–512 (2007). 10.1016/j.neures.2006.12.007 17250915

[43] Schecklmann M. et al., “Influence of muscle activity on brain oxygenation during verbal fluency assessed with functional near-infrared spectroscopy,” Neuroscience 171(2), 434–442 (2010). 10.1016/j.neuroscience.2010.08.072 20858532

[44] Konstantopoulos K. Giakoumettis D., “Cerebral organization for speech/language and neuroanatomy of speech/language disorders,” in Neuroimaging in Neurogenic Communication Disorders, Konstantopoulos K. Giakoumettis D., Eds., Academic Press, pp. 47–72 (2023).

[45] Paulesu E. Frith C. D. Frackowiak R. S., “The neural correlates of the verbal component of working memory,” Nature 362(6418), 342–345 (1993). 10.1038/362342a0 8455719

[46] Santosa H. et al., “The NIRS Brain AnalyzIR Toolbox,” Algorithms 11(5), 73 (2018).1748-7188 10.3390/a11050073 38957522 PMC11218834

[47] Lancaster J. L. et al., “Automated Talairach atlas labels for functional brain mapping,” Hum. Brain Mapp. 10(3), 120–131 (2000). HBRME7 1065-9471 10.1002/1097-0193(200007)10:3<120::AID-HBM30>3.0.CO;2-8 10912591 PMC6871915

[48] Meidenbauer K. L. et al., “Load-dependent relationships between frontal fNIRS activity and performance: a data-driven PLS approach,” NeuroImage 230, 117795 (2021). NEIMEF 1053-8119 10.1016/j.neuroimage.2021.117795 33503483 PMC8145788

[49] Hocke L. M. et al., “Automated processing of fNIRS data—a visual guide to the pitfalls and consequences,” Algorithms 11(5), 67 (2018).1748-7188 10.3390/a11050067 30906511 PMC6428450

[50] Strangman G. Franceschini M. A. Boas D. A., “Factors affecting the accuracy of near-infrared spectroscopy concentration calculations for focal changes in oxygenation parameters,” NeuroImage 18(4), 865–879 (2003). NEIMEF 1053-8119 10.1016/S1053-8119(03)00021-1 12725763

[51] Barker J. W. Aarabi A. Huppert T. J., “Autoregressive model based algorithm for correcting motion and serially correlated errors in fNIRS,” Biomed. Opt. Express 4(8), 1366–1379 (2013). BOEICL 2156-7085 10.1364/BOE.4.001366 24009999 PMC3756568

[52] Mukli P. et al., “Impaired neurovascular coupling and increased functional connectivity in the frontal cortex predict age-related cognitive dysfunction,” Adv. Sci. 11(10), e2303516 (2024). 10.1002/advs.202303516 PMC10962492 38155460

[53] Robinson G. et al., “The differing roles of the frontal cortex in fluency tests,” Brain 135(7), 2202–2214 (2012). BRAIAK 0006-8950 10.1093/brain/aws142 22669082 PMC3381725

[54] Shapira-Lichter I. et al., “Portraying the unique contribution of the default mode network to internally driven mnemonic processes,” Proc. Natl. Acad. Sci. U. S. A. 110(13), 4950–4955 (2013). 10.1073/pnas.1209888110 23479650 PMC3612673

[55] Gruenewald P. J. Lockhead G. R., “The free recall of category examples,” J. Exp. Psychol. Hum. Learn. Mem. 6(3), 225–240 (1980). JPHMD8 0096-1515 10.1037/0278-7393.6.3.225

[56] Klein A. P. et al., “Imaging of cortical and white matter language processing,” Semin. Ultrasound CT MR 36(3), 249–259 (2015). 10.1053/j.sult.2015.05.011 26233859

[57] Perret E., “The left frontal lobe of man and the suppression of habitual responses in verbal categorical behaviour,” Neuropsychologia 12(3), 323–330 (1974). NUPSA6 0028-3932 10.1016/0028-3932(74)90047-5 4421777

[58] Mummery C. J. et al., “Generating ‘tiger’ as an animal name or a word beginning with T: differences in brain activation,” Proc. R. Soc. B Biol. Sci. 263(1373), 989–995 (1996). 10.1098/rspb.1996.0146 8805836

[59] Troyer A. K. et al., “Clustering and switching on verbal fluency: the effects of focal frontal- and temporal-lobe lesions,” Neuropsychologia 36(6), 499–504 (1998). NUPSA6 0028-3932 10.1016/S0028-3932(97)00152-8 9705059

[60] Ramier A. M. Hécaen H., “Rôle respectif des atteintes frontales et de la latéralisation lésionnelle dans les déficits de la ‘fluence verbale’,” Rev. Neurol. 123(1), 17–22 (1970). 5516326

[61] Thompson-Schill S. L. et al., “Role of left inferior prefrontal cortex in retrieval of semantic knowledge: a reevaluation,” Proc. Natl. Acad. Sci. U. S. A. 94(26), 14792–14797 (1997). 10.1073/pnas.94.26.14792 9405692 PMC25116

[62] Robinson G. Blair J. Cipolotti L., “Dynamic aphasia: an inability to select between competing verbal responses?,” Brain 121(1), 77–89 (1998). BRAIAK 0006-8950 10.1093/brain/121.1.77 9549489

[63] Bisconti S. et al., “Functional near-infrared spectroscopy reveals heterogeneous patterns of language lateralization over frontopolar cortex,” Neurosci. Res. 73(4), 328–332 (2012). 10.1016/j.neures.2012.05.013 22698777

[64] Rudebeck P. H. Murray E. A., “The orbitofrontal oracle: cortical mechanisms for the prediction and evaluation of specific behavioral outcomes,” Neuron 84(6), 1143–1156 (2014). NERNET 0896-6273 10.1016/j.neuron.2014.10.049 25521376 PMC4271193

[65] Mansouri F. A. et al., “Managing competing goals—a key role for the frontopolar cortex,” Nat. Rev. Neurosci. 18(11), 645–657 (2017). NRNAAN 1471-003X 10.1038/nrn.2017.111 28951610

[66] Gourovitch M. L. et al., “A comparison of rCBF patterns during letter and semantic fluency,” Neuropsychology 14(3), 353–360 (2000). NEUPEG 10.1037/0894-4105.14.3.353 10928738

[67] Riello M. et al., “Neural correlates of letter and semantic fluency in primary progressive aphasia,” Brain Sci. 12(1), 1 (2021). 10.3390/brainsci12010001 35053745 PMC8773895

[68] Vonk J. M. J. et al., “Letter and category fluency performance correlates with distinct patterns of cortical thickness in older adults,” Cereb. Cortex 29(6), 2694–2700 (2019). 53OPAV 1047-3211 10.1093/cercor/bhy138 29893804 PMC6519688

[69] Vigneau M. et al., “Meta-analyzing left hemisphere language areas: phonology, semantics, and sentence processing,” NeuroImage 30(4), 1414–1432 (2006). NEIMEF 1053-8119 10.1016/j.neuroimage.2005.11.002 16413796

[r70] Baddeley A., “Working memory: looking back and looking forward,” Nat. Rev. Neurosci. 4(10), 829–839 (2003). NRNAAN 1471-003X 10.1038/nrn1201 14523382

[71] Deschamps I. Baum S. R. Gracco V. L., “On the role of the supramarginal gyrus in phonological processing and verbal working memory: evidence from rTMS studies,” Neuropsychologia 53, 39–46 (2014). NUPSA6 0028-3932 10.1016/j.neuropsychologia.2013.10.015 24184438

[72] Hartwigsen G. et al., “Phonological decisions require both the left and right supramarginal gyri,” Proc. Natl. Acad. Sci. U. S. A. 107(38), 16494–16499 (2010). 10.1073/pnas.1008121107 20807747 PMC2944751

[r73] Hartwigsen G. et al., “Dissociating parieto-frontal networks for phonological and semantic word decisions: a condition-and-perturb TMS study,” Cereb. Cortex 26(6), 2590–2601 (2016). 53OPAV 1047-3211 10.1093/cercor/bhv092 25953770

[74] Pillay S. B. et al., “Cerebral localization of impaired phonological retrieval during rhyme judgment,” Ann. Neurol. 76(5), 738–746 (2014). 10.1002/ana.24266 25164766 PMC4214892

[75] Hickok G. Poeppel D., “The cortical organization of speech processing,” Nat. Rev. Neurosci. 8(5), 393–402 (2007). NRNAAN 1471-003X 10.1038/nrn2113 17431404

[76] Hickok G. Okada K. Serences J. T., “Area Spt in the human planum temporale supports sensory-motor integration for speech processing,” J. Neurophysiol. 101(5), 2725–2732 (2009). JONEA4 0022-3077 10.1152/jn.91099.2008 19225172

[77] Reuter-Lorenz P. A. Cappell K. A., “Neurocognitive aging and the compensation hypothesis,” Curr. Dir. Psychol. Sci. 17(3), 177–182 (2008). CDPSE8 0963-7214 10.1111/j.1467-8721.2008.00570.x

[78] Biesbroek J. M. et al., “Anatomy of phonemic and semantic fluency: a lesion and disconnectome study in 1231 stroke patients,” Cortex 143, 148–163 (2021). 10.1016/j.cortex.2021.06.019 34450565

[79] Davidson R. J., “Anterior cerebral asymmetry and the nature of emotion,” Brain Cogn. 20(1), 125–151 (1992). 10.1016/0278-2626(92)90065-T 1389117

[80] Harmon-Jones E. Gable P. A. Peterson C. K., “The role of asymmetric frontal cortical activity in emotion-related phenomena: a review and update,” Biol. Psychol. 84(3), 451–462 (2010). 10.1016/j.biopsycho.2009.08.010 19733618

[81] Smith E. E. et al., “Assessing and conceptualizing frontal EEG asymmetry: an updated primer on recording, processing, analyzing, and interpreting frontal alpha asymmetry,” Int. J. Psychophysiol. 111, 98–114 (2017). IJPSEE 0167-8760 10.1016/j.ijpsycho.2016.11.005 27865882 PMC6449497

[82] Peterson C. K. Harmon-Jones E., “Circadian and seasonal variability of resting frontal EEG asymmetry,” Biol. Psychol. 80(3), 315–320 (2009). 10.1016/j.biopsycho.2008.11.002 19056459

