# Supplementary material for: Distinct neural signatures of phonemic and semantic verbal fluency: a double dissociation in cortical activation and functional connectivity revealed by fNIRS
Source: Neurophotonics. 2026 May 12;13(2):025009. doi: 10.1117/1.NPh.13.2.025009 (PMC13167064; doi:10.1117/1.NPh.13.2.025009)
Supplement: Supplementary file 1 [file NPh_013_025009_SD001.pdf]

Supplementary Material 1

Stroop task

To assess participants' inhibitory control, a behavioral version of the color-word Stroop task [41] was administered. Two neutral blocks and two incongruent blocks were presented in a pseudo-randomized order, the neutral condition involved matching the color of a displayed square (red, green, or blue) to corresponding labeled keys on a response pad. In the incongruent condition, participants identified the ink color of a printed color word while ignoring the word's meaning. They were instructed to respond as quickly and accurately as possible. Each task block consisted of 40 color words, which were displayed in a randomized order. The trials started with a 500 ms fixation symbol, followed by the stimulus for 500 ms and a 1500 ms inter-stimulus interval. Participants had 2000 ms to respond to each stimulus.

Reaction time (RT) and accuracy were recorded for both conditions. The Stroop ratio was calculated by dividing the Stroop difference (mean incongruent – mean neutral) by the mean of neutral and incongruent trials. A smaller Stroop ratio indicates better inhibitory control.

N-back task

A visual N-back task consisted of sessions of various difficulties was used to assess participants' Updating function.Two 0-back blocks, two 1-back blocks, and two 2-back blocks were presented in a pseudorandom order, each containing the digits 0–9.

During each task block, participants had to continuously remember a series of digits that were displayed on the screen, presented in a randomized sequence, shown as a stimulus for 1700 ms, and followed by a 500 ms fixation symbol. Participants had 2200 ms to respond to each stimulus. Each task block contained a total of 20 digits, including 5 target stimuli and the remaining non-target stimuli. Participants were requested to press the left key for target stimuli and the right key for non-target stimuli. The nback task requires participants to react when a stimulus is a target, the same as the n-th digit before the current stimulus letter. In case of 0-back, the target stimulus was “5”.

RT was defined as the average time required to complete a single trial. Accuracy was calculated as the number of correct responses to target stimuli divided by the total number of target stimuli.

More-odd-shifting task

The More-odd-shifting task was used to assess participants’ shifting function, in which two “More” blocks, two “Odd” blocks, and two “Shifting” blocks were presented in a pseudorandom order. In the “More” block, participants evaluated whether red numbers (1–4, 6–9) were greater than 5, pressing the left key for "yes" and the right key for "no." In the “Odd” block, participants determined whether green numbers were odd, responding with the left key for odd and the right key for even. The “Shifting” task integrated both tasks, requiring participants to respond based on color cues. Each task block consisted of 15 trials, Each trial began with a colored

number presented for 1500 ms, followed by a fixation symbol displayed for 500 ms. Participants had 2000 ms to respond to each stimulus.

The final measure of participants' shfiting ability was calculated as the difference between the average reaction time in the shifting condition and the average reaction time in the non-shifting conditions.

Behavioral results

Reaction Time (RT)

Due to violations of the normality assumption (Shapiro–Wilk tests, $p < .05$), non-parametric tests were used to analyze reaction time (RT).

For the Stroop task, RTs were significantly longer in the incongruent condition compared to the neutral condition, as shown by a Wilcoxon signed-rank test ($V = 3$, $p < .001$).

For the n-back task, a Friedman test revealed a significant main effect of condition, $\chi^2(2) = 131.0$, $p < .001$. Post hoc Wilcoxon signed-rank tests showed that RT increased significantly with task difficulty: 1-back vs. 0-back ($V = 414$, $p < .001$), 2-back vs. 0-back ($V = 0$, $p < .001$), and 2-back vs. 1-back ($V = 1$, $p < .001$).

For the more-odd shifting task, the Friedman test indicated a significant effect of condition, $\chi^2(2) = 109.0$, $p < .001$. Wilcoxon signed-rank tests showed that RTs were significantly longer in odd compared to more ($V = 404$, $p < .001$), shifting compared to more ($V = 19$, $p < .001$), and shifting compared to odd ($V = 27$, $p < .001$).

Accuracy (ACC)

A similar non-parametric approach was adopted for accuracy (ACC) data.

For the Stroop task, accuracy was significantly lower in the incongruent compared to the neutral condition ($V = 975.5$, $p = .009$), according to the Wilcoxon signed-rank test.

In the n-back task, the Friedman test revealed a significant main effect of condition, $\chi^2(2) = 72.9$, $p < .001$. Wilcoxon signed-rank tests showed significantly lower accuracy in 1-back vs. 0-back ($V = 912$, $p < .001$), 2-back vs. 0-back ($V = 1596$, $p < .001$), and 2-back vs. 1-back ($V = 1476$, $p < .001$), all Bonferroni-corrected.

In the more-odd shifting task, accuracy differed significantly by condition, $\chi^2(2) = 79.8$, $p < .001$ (Friedman test). Wilcoxon comparisons revealed significant accuracy decreases across all pairs: odd vs. more ($V = 1496$, $p < .001$), shifting vs. more ($V = 2547$, $p < .001$), and shifting vs. odd ($V = 2122$, $p < .001$), Bonferroni corrected.

Supplementary Material 2

Analysis of task-based activation

Task-based activation was analyzed using a pipeline based on General Linear Model (GLM) approach, which uses autoregressive iteratively reweighted leastsquares model (AR-IRLS). In this process, to mitigate serially correlated errors arising from systemic physiology and motion artifacts, an autoregressive model-based algorithm was applied for pre-whitening, while robust weighted regression iteratively down-weighted outliers caused by motion artifacts [51]. Following this process, first-level β estimates were entered into a group mixed-effects model [46] with Condition as fixed and Subject as random effect, defined by the Wilkinson-Rogers formula: '$\beta \sim -1 + \text{condition} + (1|\text{Subject})$', the results were used to compute group-level contrasts between task conditions for each channel by defining t-contrasts.

Benjamini–Hochberg FDR was used to correct for multiple comparisons at the group level.

Contrasting Task-Based Activation of pVFT and sVFT

For the statistical analysis, only oxyhemoglobin (HbO) signals were included. To systematically analyze the activation differences between pVFT and sVFT at the ROI level, and to examine hemispheric lateralization, a three-way repeated-measures ANOVA was conducted. The model included ROI, Hemisphere, and Task as within-subject factors, with the beta (β) coefficient from the GLM as the dependent variable. The investigation of lateralization was motivated by prior findings

suggesting that left frontal lesions impair VFT performance more than right frontal lesions [53], and that fMRI studies have similarly demonstrated left-lateralized activation during VFT [56]. This analysis also aimed to test whether the degree of lateralization differs between pVFT and sVFT.

To further interpret the significant interaction effects observed in the ROI × Hemisphere × Task ANOVA, a series of region-specific analyses were conducted at the group level to further characterize the activation and lateralization patterns associated with pVFT and sVFT.

First, To examine activation differences between pVFT and sVFT within each ROI, individual-level activation data were first averaged using the roiAverage function . A mixed-effects model was then applied with the formula '$\beta \sim -1 +$ condition + (1|Subject)', and t-contrasts were conducted to compare activation between pVFT and sVFT conditions.

Second, to assess the degree of lateralization within each ROI under the two tasks, we employed the roi_math function to define left–right hemisphere contrasts (e.g., left_BA46 – right_BA46), and applied these contrasts to the group-level activation results.

Third, to compare the lateralization levels between pVFT and sVFT across ROIs, we conducted paired t-contrasts on the outputs of the roi_math procedure, using the defined left–right differences for each task condition.

Analysis of Funtional Connectivity

Functional connectivity (FC) was assessed to quantify the temporal synchrony of brain activation patterns between all channel pairs for both pVFT and sVFT. For this purpose, Pearson correlation coefficients (r) were computed. To enhance the robustness of these correlation estimates and to account for physiological noise and temporal autocorrelation inherent in fNIRS data, an AR-whitened correlation method was utilized. Specifically, the nirs.sFC.ar_corr function was applied, which models and removes autoregressive components from the time-series data and incorporates robust estimation techniques to mitigate the influence of outliers [46]. Because each condition contained two blocks, we computed r per block, Fisher-Z transformed, averaged Z within condition, and inverse-transformed to obtain one FC value (r) per participant per condition.

Functional connectivity was analyzed using the MixedEffectsConnectivity function, which utilizes a mixed-effects regression model similar to our activation analysis pipeline. This approach was used to compute group-level adjacency matrices for pVFT, sVFT, and the direct contrast between them. We specified two different models: the formula ‘R ~ -1 + cond + (1|Subject)’ with dummyCoding = 'full' was used to derive task-specific matrices, while the formula ‘R ~ 1 + cond + (1|Subject)’ with dummyCoding = 'reference' was used to compute the contrast matrix comparing the two conditions. To identify significant connections, these adjacency matrices underwent a thresholding step. Any values that did not meet the criteria of q_value <

0.01 and z_value > 0 were set to zero.

Graph theoretical analysis was used to characterize the topology of functional brain networks, treating each measurement channel as a node and each functional connection as an edge (or link). Binary and weighted graph theoretical parameters were calculated to reveal the number and strength of connections, respectively.

Using the individual-level adjacency matrices (where values not meeting the criteria of q-value < 0.01 and z-value > 0 were set to zero), we computed weighted local node degrees and binarized local node degrees for each participant and channel. The weighted local node degree represents the average connection strength (Pearson correlation value) of a node's connections. The binarized local node degree represents the average of the binarized Pearson coefficients for each node.

Subsequently, using the same individual-level adjacency matrices, we computed the weighted global node degree and binarized global node degree for each of the two VFTs. These global degrees were calculated as the average of the local node degrees across all nodes.

Contrasting Functional Connectivity of pVFT and sVFT

To examine functional connectivity differences between the two VFTs across various connection types, we conducted a series of LME analyses.

We averaged Fisher Z-transformed connectivity within each connection type per participant to obtain one Z-value per type, which served as the dependent variable in the LME models.

Connection types were categorized at three hierarchical levels, and a separate

LME model was conducted for each category:

- LME for Hemispheric Connectivity: This analysis included intra-left hemisphere, intra-right hemisphere, and inter-hemispheric connections.
- LME for Within-ROI Connectivity: This focused on connections within each defined ROI.
- LME for Inter-Regional Connectivity: This analysis examined the pairwise connections between the six core regions: the bilateral PFC, MTG, and SMG.

For each connection classification, we fit a separate LME model with the formula 'Z ~ Condition × ConnectionType + (1 | Subject)' Here, Condition (phonemic vs. semantic) and ConnectionType were included as fixed effects, and Subject as a random intercept to account for between-subject variability.

To further examine the differences between the phonemic and semantic conditions within each connection type, we conducted post hoc pairwise comparisons using the emmeans package in R. We specified 'pairwise ~ Condition | ConnectionType' to estimate the simple effects of Condition within each connection type. The contrast "phonemic - semantic" was retained for reporting.

To explore the differences in both node connection strength and connection number between the two VFTs, we analyzed both local and global graph theoretical parameters. For the local node degrees (weighted and binarized), we used a LME model for each channel. We fit the model 'Degree ~ -1 + Condition + (1 | Subject)' to compute the beta (β) values for each VFT condition, then fit the model 'Degree ~ Condition + (1 | Subject)' to calculate the t-contrast values for the direct comparison

between the two VFTs. Both the β values and the t-contrast values (with $p < 0.05$) were then mapped onto the 10-20 map. For the global node degrees, we performed paired-samples t-tests on the individual-level weighted and binarized global node degrees to compare the two VFT conditions.

Supplementary Data File S1

Channal	ROI	Hemisphere
S1D1	MTG	Right
S1D6	MTG	Right
S2D1	MTG	Right
S2D2	BA46	Right
S2D2	IFG	Right
S2D7	BA46	Right
S2D7	IFG	Right
S3D2	BA46	Right
S3D2	OFC	Right
S3D3	OFC	Right
S3D8	BA46	Right
S4D3	OFC	Left
S4D4	BA46	Left
S4D4	OFC	Left
S4D9	BA46	Left
S5D4	BA46	Left
S5D4	IFG	Left
S5D5	MTG	Left
S5D10	BA46	Left
S5D10	IFG	Left
S6D5	MTG	Left
S6D11	MTG	Left
S7D1	MTG	Right
S7D6	MTG	Right
S7D6	SMG	Right
S7D7	BA9	Right
S7D7	IFG	Right
S8D2	BA46	Right
S8D2	IFG	Right
S8D7	BA46	Right
S8D7	BA9	Right
S8D7	IFG	Right
S8D8	BA46	Right
S8D8	BA9	Right
S8D13	BA46	Right
S8D13	BA9	Right
S8D13	IFG	Right
S9D3	OFC	Left
S9D8	BA9	Right
S9D9	BA9	Left
S9D14	BA9	Left

S10D4	BA46	Left
S10D4	IFG	Left
S10D9	BA46	Left
S10D9	BA9	Left
S10D10	BA46	Left
S10D10	BA9	Left
S10D10	IFG	Left
S10D15	BA46	Left
S10D15	BA9	Left
S10D15	IFG	Left
S11D5	MTG	Left
S11D10	BA9	Left
S11D10	IFG	Left
S11D11	MTG	Left
S11D11	SMG	Left
S12D12	SMG	Right
S13D8	BA46	Right
S13D8	BA9	Right
S13D13	BA9	Right
S13D14	BA9	Right
S14D9	BA46	Left
S14D9	BA9	Left
S14D14	BA9	Left
S14D15	BA9	Left
S15D12	SMG	Right
S16D12	SMG	Right
S17D12	SMG	Right
S18D16	SMG	Left
S19D16	SMG	Left
S20D16	SMG	Left
S21D16	SMG	Left

Supplementary Data File S2

source	detector	cond	beta	se	tstat	dfe	p	q
1	1	phonemic	16.093147	1.9387147	8.3009363	154	4.90E-14	1.74E-13
1	6	phonemic	12.661554	1.7383931	7.2834814	154	1.57E-11	4.44E-11
2	1	phonemic	15.371581	1.6252623	9.4579075	154	5.08E-17	3.75E-16
2	2	phonemic	16.982201	1.4284128	11.888862	154	1.54E-23	2.47E-22
2	7	phonemic	12.386551	1.3414193	9.2339139	154	1.96E-16	1.04E-15
3	2	phonemic	19.09163	1.5805972	12.078745	154	4.71E-24	9.05E-23
3	3	phonemic	11.307216	1.3744387	8.2267879	154	7.54E-14	2.58E-13
3	8	phonemic	8.6142107	1.2973424	6.6398899	154	5.08E-10	1.28E-09
4	3	phonemic	12.790225	1.3704225	9.3330521	154	1.08E-16	6.47E-16
4	4	phonemic	22.947945	1.5128601	15.168584	154	2.26E-32	1.54E-30
4	9	phonemic	11.85719	1.3952417	8.4983051	154	1.55E-14	6.19E-14
5	4	phonemic	21.699159	1.435971	15.11114	154	3.22E-32	1.54E-30
5	5	phonemic	18.447883	1.7128782	10.770108	154	1.63E-20	1.96E-19
5	10	phonemic	19.373793	1.4395187	13.458521	154	8.63E-28	2.76E-26
6	5	phonemic	15.198705	1.9422711	7.8252229	154	7.56E-13	2.27E-12
6	11	phonemic	14.650374	1.6898722	8.6695163	154	5.65E-15	2.36E-14
7	1	phonemic	13.851619	1.5696924	8.8244163	154	2.26E-15	1.09E-14
7	6	phonemic	10.854748	1.581422	6.8639166	154	1.54E-10	4.00E-10
7	7	phonemic	11.98539	1.3651111	8.7797912	154	2.95E-15	1.35E-14
8	2	phonemic	14.618424	1.5362991	9.5153502	154	3.59E-17	2.87E-16
8	7	phonemic	13.52425	1.4453532	9.3570553	154	9.33E-17	5.97E-16
8	8	phonemic	11.998093	1.2800223	9.3733464	154	8.46E-17	5.80E-16
8	13	phonemic	9.4141657	1.3232526	7.1144129	154	3.98E-11	1.06E-10
9	3	phonemic	7.5383416	1.2688542	5.9410619	154	1.82E-08	4.06E-08
9	8	phonemic	8.7959326	1.1747828	7.4872842	154	5.08E-12	1.48E-11
9	9	phonemic	9.9107727	1.0635356	9.3187036	154	1.18E-16	6.64E-16
9	14	phonemic	11.175137	1.2532287	8.9170769	154	1.30E-15	6.59E-15
10	4	phonemic	20.414381	1.5608725	13.078827	154	9.18E-27	2.20E-25
10	9	phonemic	17.308609	1.4690341	11.782306	154	3.00E-23	4.11E-22
10	10	phonemic	15.148003	1.4497831	10.448461	154	1.19E-19	1.14E-18
10	15	phonemic	11.388409	1.3883542	8.2028121	154	8.66E-14	2.87E-13
11	5	phonemic	13.926242	1.7724616	7.8570063	154	6.31E-13	1.95E-12
11	10	phonemic	16.548353	1.5597955	10.60931	154	4.41E-20	4.70E-19
11	11	phonemic	10.847697	1.6376524	6.6239314	154	5.52E-10	1.36E-09
12	12	phonemic	9.9158616	1.5251804	6.5014353	154	1.05E-09	2.52E-09
13	8	phonemic	5.743388	1.1256939	5.1020867	154	9.76E-07	1.91E-06
13	13	phonemic	7.5994952	1.2186275	6.2361099	154	4.12E-09	9.42E-09
13	14	phonemic	6.8883702	1.2319703	5.5913444	154	1.00E-07	2.09E-07
14	9	phonemic	8.3100254	1.1680319	7.1145538	154	3.97E-11	1.06E-10
14	14	phonemic	6.6488688	1.1319395	5.8738727	154	2.54E-08	5.53E-08
14	15	phonemic	6.9977396	1.3640817	5.1300003	154	8.60E-07	1.72E-06

15	12	phonemic	9.4477482	1.6383935	5.7664709	154	4.29E-08	9.16E-08
16	12	phonemic	15.851793	1.6647865	9.5218176	154	3.45E-17	2.87E-16
17	12	phonemic	13.353049	1.6727091	7.9828874	154	3.07E-13	9.84E-13
18	16	phonemic	12.292379	1.460153	8.4185559	154	2.47E-14	9.48E-14
19	16	phonemic	13.720819	1.6384663	8.3741841	154	3.20E-14	1.18E-13
20	16	phonemic	9.9034899	1.5748111	6.2886843	154	3.15E-09	7.38E-09
21	16	phonemic	14.161635	1.6296949	8.6897462	154	5.02E-15	2.19E-14
1	1	semantic	13.592184	1.9424248	6.9975344	154	7.51E-11	3.43E-10
1	6	semantic	11.300757	1.7311419	6.5279209	154	9.14E-10	3.37E-09
2	1	semantic	11.947011	1.6224247	7.3636765	154	1.01E-11	5.39E-11
2	2	semantic	9.7866622	1.4259834	6.8630969	154	1.55E-10	6.76E-10
2	7	semantic	6.9995505	1.3388329	5.2280987	154	5.50E-07	1.43E-06
3	2	semantic	7.7428569	1.5816822	4.8953304	154	2.45E-06	5.75E-06
3	3	semantic	6.9953461	1.3812203	5.0646127	154	1.16E-06	2.85E-06
3	8	semantic	5.6862807	1.2977219	4.3817407	154	2.17E-05	4.62E-05
4	3	semantic	8.7380177	1.3656903	6.3982427	154	1.79E-09	6.38E-09
4	4	semantic	12.365141	1.5047293	8.2175184	154	7.95E-14	5.09E-13
4	9	semantic	13.749216	1.391908	9.8779633	154	3.95E-18	6.32E-17
5	4	semantic	13.575022	1.4322745	9.4779475	154	4.50E-17	4.80E-16
5	5	semantic	15.102865	1.7168826	8.7966788	154	2.67E-15	2.56E-14
5	10	semantic	11.327205	1.4347119	7.8951075	154	5.08E-13	3.05E-12
6	5	semantic	11.906462	1.9375478	6.145119	154	6.55E-09	2.03E-08
6	11	semantic	11.490177	1.6942986	6.7816714	154	2.39E-10	9.57E-10
7	1	semantic	8.89199	1.5815106	5.6224663	154	8.62E-08	2.51E-07
7	6	semantic	6.4883466	1.5786758	4.1099929	154	6.41E-05	0.0001207
7	7	semantic	9.8581679	1.3750076	7.169537	154	2.94E-11	1.49E-10
8	2	semantic	6.4670292	1.5269417	4.2352823	154	3.91E-05	7.83E-05
8	7	semantic	6.5720027	1.4431977	4.5537785	154	1.06E-05	2.43E-05
8	8	semantic	7.0853175	1.2824451	5.5248507	154	1.37E-07	3.77E-07
8	13	semantic	5.6705004	1.3177804	4.3030692	154	2.98E-05	6.22E-05
9	3	semantic	4.5424129	1.2686051	3.5806358	154	0.0004589	0.0007868
9	8	semantic	5.3019857	1.1715468	4.5256286	154	1.20E-05	2.67E-05
9	9	semantic	8.7407074	1.0598923	8.2467881	154	6.71E-14	4.60E-13
9	14	semantic	7.9773294	1.2503796	6.3799263	154	1.97E-09	6.76E-09
10	4	semantic	11.959674	1.5650743	7.6416012	154	2.14E-12	1.21E-11
10	9	semantic	12.913066	1.4722146	8.7711846	154	3.10E-15	2.71E-14
10	10	semantic	14.581639	1.4466798	10.079382	154	1.15E-18	2.21E-17
10	15	semantic	7.7123427	1.3890373	5.5522935	154	1.21E-07	3.41E-07
11	5	semantic	7.4955039	1.7756464	4.2212819	154	4.14E-05	8.11E-05
11	10	semantic	13.094317	1.5606597	8.3902446	154	2.91E-14	2.15E-13
11	11	semantic	9.6390838	1.6397874	5.8782522	154	2.48E-08	7.45E-08
12	12	semantic	10.82101	1.5316528	7.0649234	154	5.21E-11	2.50E-10
13	8	semantic	5.8636138	1.1268446	5.2035691	154	6.15E-07	1.55E-06

13	13	semantic	4.7536258	1.2218666	3.8904621	154	0.0001485	0.000269
13	14	semantic	7.646424	1.2298304	6.2174623	154	4.53E-09	1.50E-08
14	9	semantic	9.9388737	1.1682813	8.5072606	154	1.47E-14	1.17E-13
14	14	semantic	7.4018532	1.129245	6.5546918	154	7.94E-10	3.05E-09
14	15	semantic	3.4559439	1.3609796	2.5393061	154	0.0120988	0.0190408
15	12	semantic	11.195686	1.6385519	6.8326709	154	1.82E-10	7.61E-10
16	12	semantic	20.18418	1.6616204	12.147287	154	3.07E-24	2.95E-22
17	12	semantic	19.704132	1.6802109	11.727178	154	4.23E-23	2.03E-21
18	16	semantic	16.360191	1.4524982	11.263485	154	7.61E-22	1.83E-20
19	16	semantic	15.699242	1.6452827	9.5419723	154	3.05E-17	3.66E-16
20	16	semantic	15.07091	1.5713833	9.590855	154	2.27E-17	3.11E-16
21	16	semantic	18.722282	1.6284747	11.496821	154	1.78E-22	5.69E-21
1	1	phonemic -	2.5009625	2.2291942	1.1219132	154	0.2636456	0.346712
1	6	phonemic -	1.3607968	1.8640936	0.7300045	154	0.4664964	0.5462213
2	1	phonemic -	3.4245699	1.7253639	1.9848392	154	0.0489387	0.0886436
2	2	phonemic -	7.1955392	1.4162954	5.0805356	154	1.08E-06	9.39E-06
2	7	phonemic -	5.3870001	1.3715716	3.9276114	154	0.0001291	0.0006199
3	2	phonemic -	11.348773	1.5573911	7.287041	154	1.54E-11	4.94E-10
3	3	phonemic -	4.31187	1.2667206	3.4039629	154	0.0008468	0.0029033
3	8	phonemic -	2.9279299	1.1611817	2.5215088	154	0.0127021	0.0274863
4	3	phonemic -	4.0522073	1.2535405	3.2326098	154	0.0015006	0.004647
4	4	phonemic -	10.582804	1.4126981	7.4912005	154	4.97E-12	2.38E-10
4	9	phonemic -	-1.892026	1.3334992	-1.418843	154	0.1579654	0.2328087
5	4	phonemic -	8.1241364	1.3659948	5.947414	154	1.76E-08	3.38E-07
5	5	phonemic -	3.3450183	1.9224289	1.7399959	154	0.0838575	0.1413232
5	10	phonemic -	8.0465887	1.5355044	5.2403553	154	5.20E-07	4.99E-06
6	5	phonemic -	3.2922425	2.2086701	1.4905995	154	0.1381121	0.2138509
6	11	phonemic -	3.1601977	1.8165248	1.7396943	154	0.0839106	0.1413232
7	1	phonemic -	4.9596292	1.7092117	2.9017057	154	0.0042558	0.0120163
7	6	phonemic -	4.3664019	1.7151252	2.5458211	154	0.0118845	0.0274863
7	7	phonemic -	2.1272224	1.3912144	1.52904	154	0.128306	0.2060275
8	2	phonemic -	8.1513951	1.4678897	5.5531388	154	1.20E-07	1.65E-06
8	7	phonemic -	6.952247	1.4809542	4.6944374	154	5.87E-06	4.33E-05
8	8	phonemic -	4.9127751	1.0464837	4.6945547	154	5.86E-06	4.33E-05
8	13	phonemic -	3.7436653	1.2084183	3.0979878	154	0.0023163	0.0069489
9	3	phonemic -	2.9959286	1.1803096	2.5382565	154	0.0121337	0.0274863
9	8	phonemic -	3.4939468	0.916268	3.8132369	154	0.000198	0.000864
9	9	phonemic -	1.1700654	0.7587064	1.5421847	154	0.1250811	0.2060275
9	14	phonemic -	3.1978075	1.2328487	2.5938362	154	0.0104068	0.0249764
10	4	phonemic -	8.4547073	1.6018724	5.2780155	154	4.37E-07	4.66E-06
10	9	phonemic -	4.3955427	1.1243958	3.9092486	154	0.0001384	0.0006327
10	10	phonemic -	0.5663636	1.2575635	0.4503658	154	0.6530801	0.6889636
10	15	phonemic -	3.6760658	1.1957228	3.0743461	154	0.0024963	0.0072619

11	5	phonemic -	6.4307377	1.9838727	3.2415072	154	0.0014574	0.004647
11	10	phonemic -	3.4540366	1.6536785	2.088699	154	0.0383801	0.0739311
11	11	phonemic -	1.2086136	1.7474672	0.6916374	154	0.4902065	0.553645
12	12	phonemic -	-0.905148	1.4784236	-0.612239	154	0.5412828	0.5972776
13	8	phonemic -	-0.120226	0.8347149	-0.144032	154	0.8856634	0.8949862
13	13	phonemic -	2.8458694	1.130606	2.5171186	154	0.012855	0.0274863
13	14	phonemic -	-0.758054	1.0783952	-0.702946	154	0.4831505	0.5521721
14	9	phonemic -	-1.628848	0.8947423	-1.820466	154	0.0706294	0.1255634
14	14	phonemic -	-0.752984	1.0101351	-0.745429	154	0.4571483	0.5462213
14	15	phonemic -	3.5417958	1.287309	2.7513174	154	0.0066479	0.0172537
15	12	phonemic -	-1.747938	1.5775627	-1.107999	154	0.2695898	0.3497381
16	12	phonemic -	-4.332387	1.6500159	-2.625664	154	0.0095206	0.0234352
17	12	phonemic -	-6.351083	1.5033715	-4.22456	154	4.08E-05	0.0002614
18	16	phonemic -	-4.067812	1.0022119	-4.058834	154	7.82E-05	0.0004172
19	16	phonemic -	-1.978423	1.6572913	-1.193769	154	0.2344039	0.3169405
20	16	phonemic -	-5.16742	1.2505851	-4.132002	154	5.88E-05	0.0003323
21	16	phonemic -	-4.560647	1.6576853	-2.751214	154	0.0066499	0.0172537

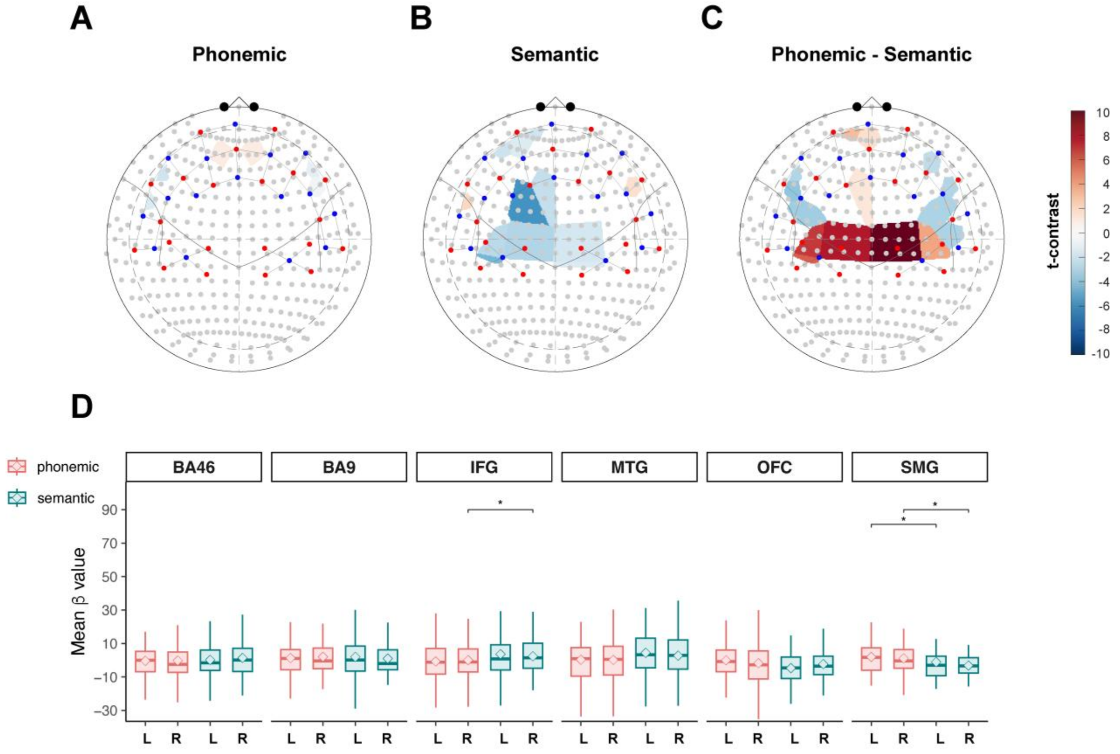

Figure S1 HbR Activation Statistics at the Channel and ROI Levels

(A;B;C) FDR-corrected t-statistics (indicated by the color-coded t-values with a cutoff at $q < 0.05$) for the task-specific HbO activation patterns of pVFT, sVFT and the contrasts between pVFT and sVFT.

(D) Boxplots of mean HbO beta values within each ROI for pVFT and sVFT. Diamonds represent mean values, and significance levels derived from the LME contrasts are indicated on the plots (*$p < 0.05$, **$p < 0.01$).

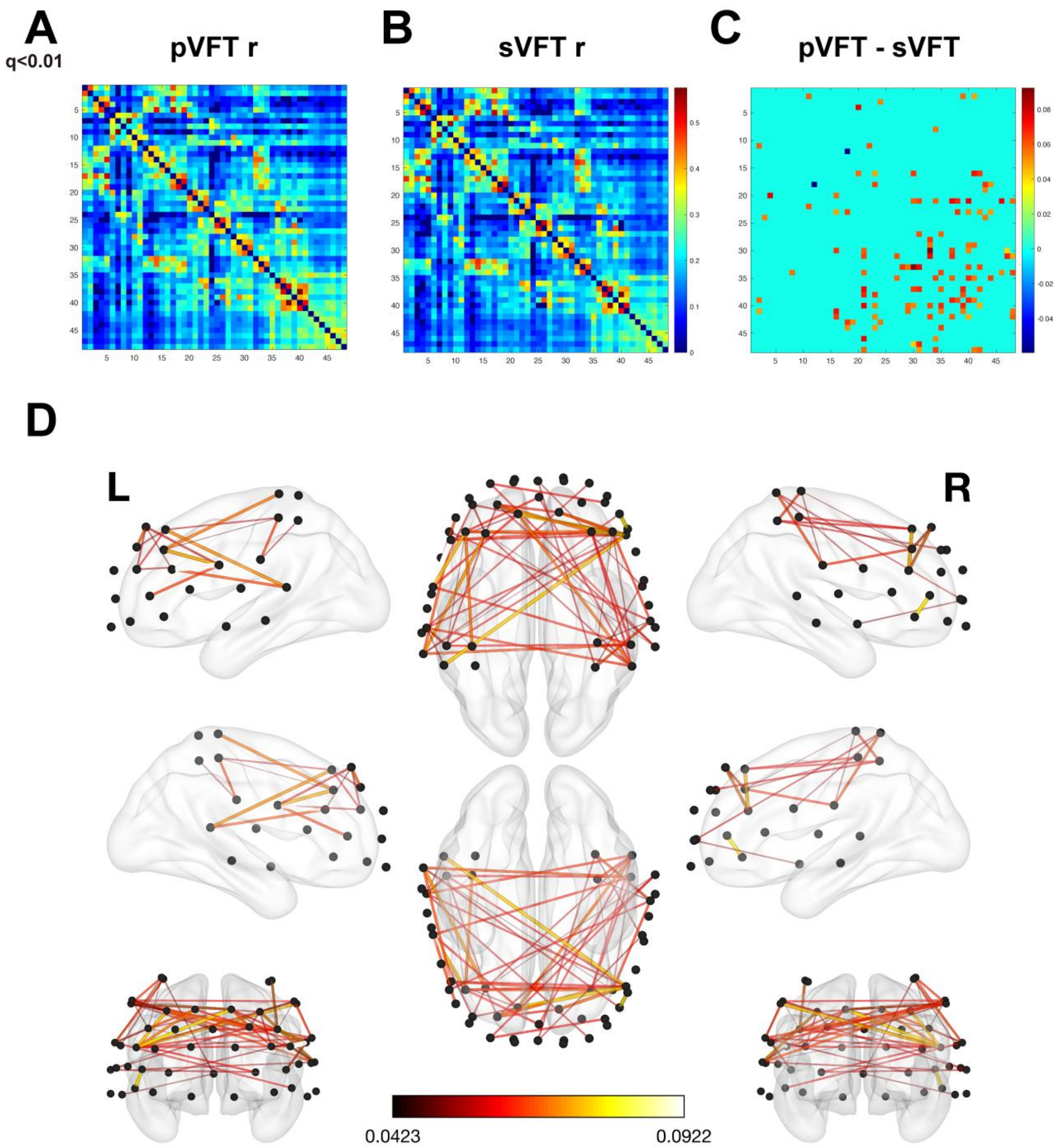

Figure S2 Adjacency matrices and FC contrast maps for HbR

(A;B;C) Color-coded group-level Pearson adjacency matrices for pVFT and sVFT, as well as their direct contrast, were computed using the Mixed Effects Connectivity function in the NIRS Toolbox. Values with q > 0.01 were set to zero. (D) Significant connectivity differences between pVFT and sVFT are projected onto the ICBM152 brain template from multiple viewing angles. Line thickness encodes the magnitude of task-related differences, and line colors follow the colormap shown in the figure.

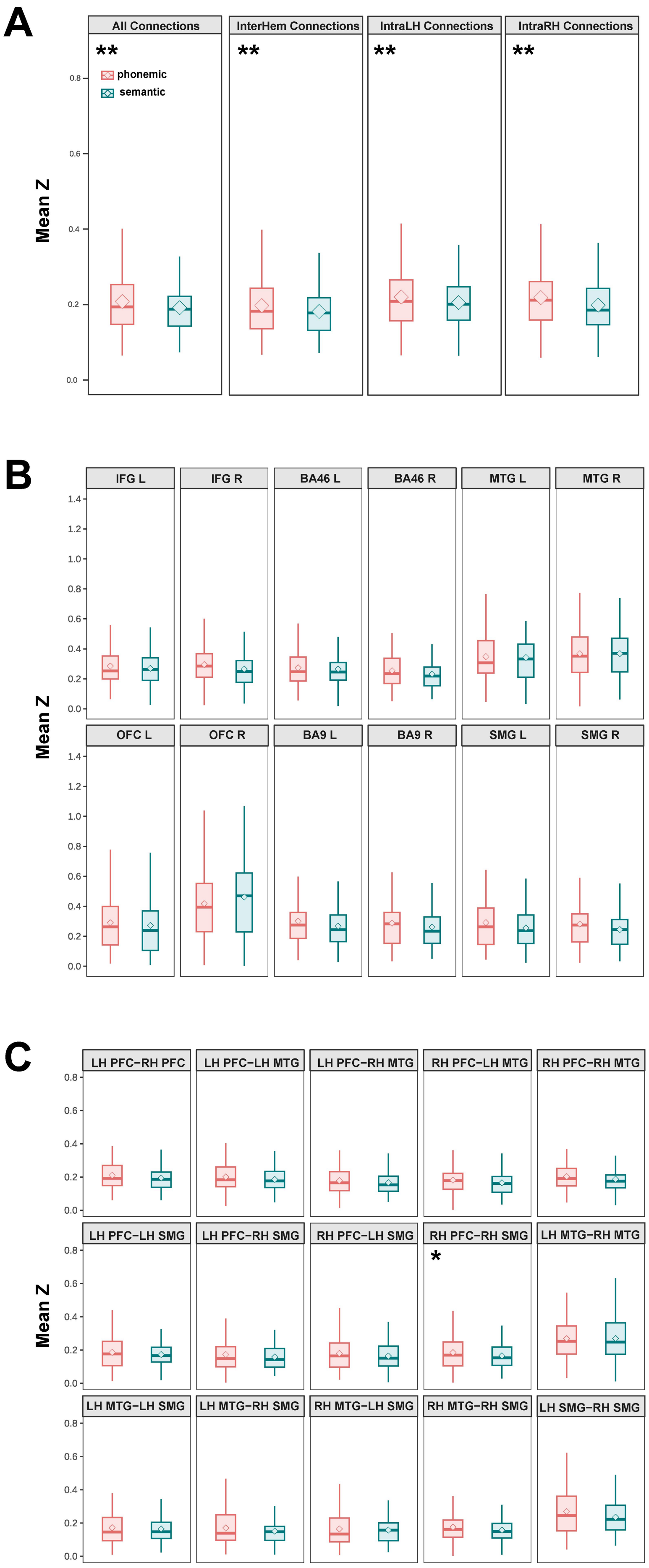
A
All Connections
InterHem Connections
IntraLH Connections
IntraRH Connections
**
phonemic
semantic
Mean Z
0.0
0.2
0.4
0.6
0.8
B
IFG L
IFG R
BA46 L
BA46 R
MTG L
MTG R
OFC L
OFC R
BA9 L
BA9 R
SMG L
SMG R
1.4
1.2
1.0
C
LH PFC–RH PFC
LH PFC–LH MTG
LH PFC–RH MTG
RH PFC–LH MTG
RH PFC–RH MTG
LH PFC–LH SMG
LH PFC–RH SMG
RH PFC–LH SMG
RH PFC–RH SMG
LH MTG–RH MTG
LH MTG–LH SMG
LH MTG–RH SMG
RH MTG–LH SMG
RH MTG–RH SMG
LH SMG–RH SMG
*

Figure S3 Differences between the two VFTs across connection types (HbR)

(A) Hemispheric Connectivity: intra-left hemisphere, intra-right hemisphere, and inter-hemispheric connections.
(B) Within-ROI Connectivity: connections confined to each defined ROI.
(C) Inter-Regional Connectivity: pairwise connections between the six regions, including the bilateral PFC, MTG, and SMG.

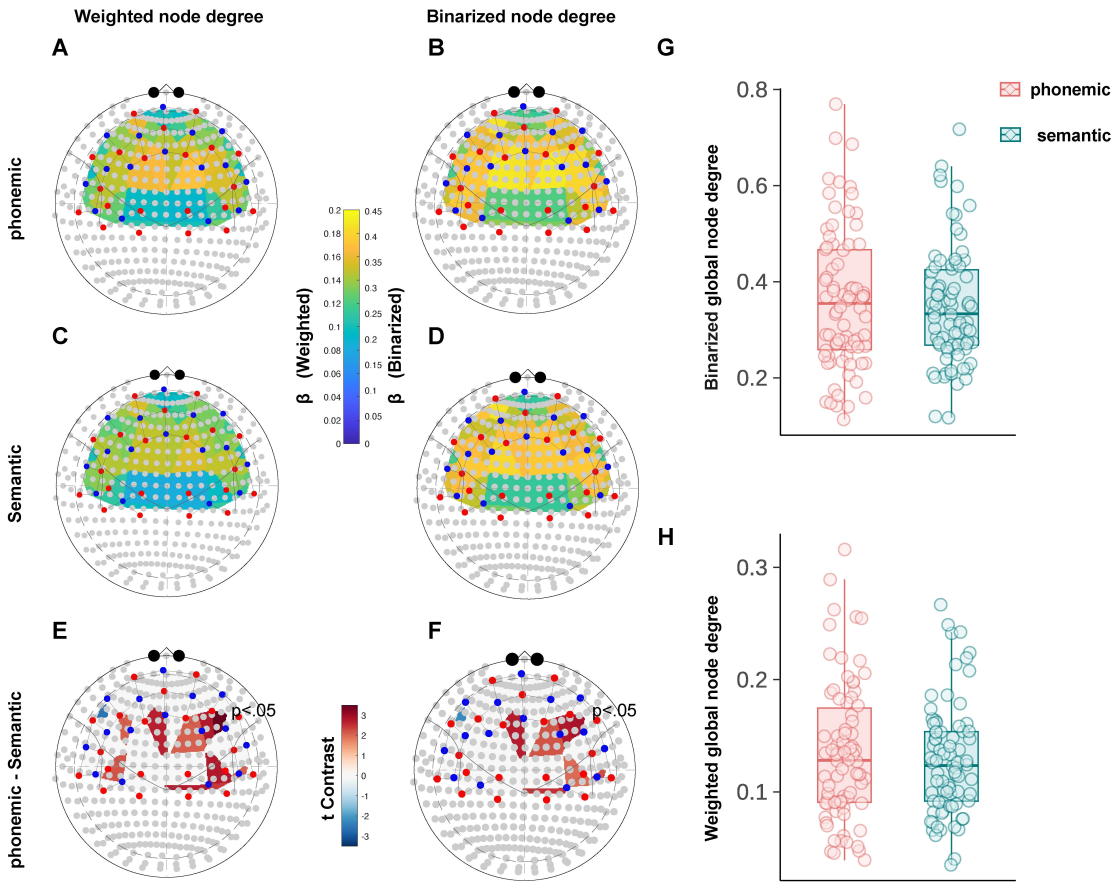

Figure S4 Graph-theoretical differences between pVFT and sVFT tasks (HbR)

Channel-level beta values of weighted and binarized local node degrees for pVFT (A;B) and sVFT (C;D), together with channel-level t-contrast results between the two tasks (E;F), mapped onto the 10–20 system. All values were derived from linear mixed-effects (LME) models. Beta values and t-contrast values were visualized using separate colorbars; further details are provided in the main text.

(G;H) Boxplots of global node degrees obtained from within-subject t-tests, (G) Binarized global node degree. (H) Weighted global node degree. In both cases, pVFT tended to show higher values than sVFT, but the difference was not statistically significant.

Supplementary Data File S3

Behavior	ROI	estimate	p.value
BCP	BA9_Right	-0.229378	0.0435799
Sub—RT	MTG_Righ	0.2125849	0.0618157
Sub—RT	BA9_Right	-0.211067	0.0637321
BCP	SMG_Righ	-0.207881	0.067914
BCP	BA46_Righ	-0.194148	0.0885439
BCP	SMG_Left	-0.189418	0.0967031
CR	OFC_Right	-0.165306	0.1480809
Cluster Size	IFG_Right	-0.158602	0.1654677
WCP	MTG_Left	0.1560292	0.1753946
Switches	SMG_Righ	0.154722	0.1761941
Switches	MTG_Righ	0.1540249	0.1781739
Switches	IFG_Right	0.151845	0.1844698
BCP	BA46_Left	-0.140682	0.2187638
Switches	SMG_Left	0.1408188	0.2187955
CR	SMG_Left	0.1313376	0.2517173
CR	SMG_Righ	0.1255854	0.2732637
CR	MTG_Righ	0.1219998	0.2873024
BCP	BA9_Left	-0.108689	0.3427873
BCP	OFC_Right	-0.10578	0.3559166
Cluster Size	MTG_Righ	-0.10549	0.3579963
Switches	BA9_Right	0.1019355	0.374513
WCP	OFC_Left	-0.099585	0.3888477
BCP	MTG_Righ	-0.098749	0.3889239
Sub—RT	BA46_Righ	-0.091516	0.4247281
Cluster Size	BA9_Left	0.0857249	0.455514
BCP	IFG_Right	-0.084662	0.4603408
WCP	OFC_Right	-0.083956	0.4678727
Sub—RT	OFC_Left	-0.082942	0.4695283
WCP	BA9_Right	-0.082865	0.4736962
CR	BA46_Left	-0.08086	0.4815812
Sub—RT	OFC_Right	-0.070272	0.5401957
CR	BA9_Left	-0.067291	0.5583034
WCP	MTG_Righ	0.066434	0.5659301
Switches	OFC_Right	-0.05958	0.6043478
BCP	OFC_Left	-0.059447	0.6044506
Switches	BA46_Righ	0.0587051	0.6096734
CR	BA46_Righ	-0.057395	0.6176884
Sub—RT	IFG_Right	0.0560832	0.6250838
Cluster Size	MTG_Left	-0.055034	0.6322502
WCP	IFG_Right	0.0532234	0.6457166
BCP	IFG_Left	-0.049634	0.6654529

Cluster Size	SMG_Left	0.0476741	0.6785192
Cluster Size	BA46_Left	0.0456382	0.6915408
Sub—RT	SMG_Right	0.0443733	0.699111
WCP	SMG_Right	-0.044666	0.6996998
Switches	BA46_Left	-0.042584	0.7112424
Switches	BA9_Left	-0.042191	0.713791
Sub—RT	MTG_Left	0.0385817	0.7368506
BCP	MTG_Left	0.0382023	0.7393458
Cluster Size	BA46_Righ	-0.033435	0.7713536
WCP	IFG_Left	-0.030996	0.7890071
Cluster Size	OFC_Right	-0.02949	0.7977218
Cluster Size	OFC_Left	0.0270364	0.8142343
WCP	BA46_Righ	0.0257376	0.8241666
Sub—RT	BA9_Left	-0.024798	0.8290633
CR	MTG_Left	-0.024808	0.8293046
WCP	BA46_Left	0.0236344	0.838332
WCP	BA9_Left	-0.023109	0.8418816
Sub—RT	SMG_Left	-0.021358	0.8524953
CR	IFG_Left	-0.017624	0.87828
Cluster Size	SMG_Right	-0.016541	0.8857111
CR	OFC_Left	-0.016382	0.8867967
CR	IFG_Right	0.0163824	0.8867967
Switches	IFG_Left	0.0139792	0.9033158
Cluster Size	IFG_Left	-0.012241	0.9152903
Switches	MTG_Left	0.011229	0.9222707
Sub—RT	IFG_Left	-0.004666	0.9676687
WCP	SMG_Left	-0.004614	0.9682332
Cluster Size	BA9_Right	0.0028959	0.9799252
CR	BA9_Right	0.0027494	0.9809403
Sub—RT	BA46_Left	0.0019348	0.9866428
Switches	OFC_Left	0.0015209	0.9894563

Supplementary Data File S4

p-values for the main effects of behavioral variables and their interaction with Condition on HbR activation levels across ROIs. No effects survived FDR correction.

	BA46_L	BA46_R	BA9_L	BA9_R	IFG_L	IFG_R	MTG_L	MTG_R	OFC_L	OFC_R	SMG_L	SMG_R
BCP	0.01	0.65	0.763	0.983	0.01	0.727	0.247	0.484	0.091	0.303	0.49	0.704
BCP × task	0.062	0.357	0.19	0.427	0.137	0.77	0.267	0.043	0.113	0.437	0.848	0.807
CR	0.373	0.597	0.574	0.269	0.34	0.163	0.266	0.392	0.292	0.235	0.506	0.524
CR × task	0.415	0.333	0.864	0.368	0.383	0.074	0.284	0.132	0.2	0.14	0.243	0.836
Cluster Size	0.741	0.222	0.844	0.121	0.801	0.953	0.701	0.357	0.14	0.513	0.068	0.687
Cluster Size × task	0.768	0.635	0.71	0.557	0.938	0.542	0.949	0.483	0.171	0.504	0.15	0.863
Sub-RT	0.305	0.949	0.851	0.841	0.263	0.614	0.49	0.658	0.431	0.812	0.229	0.025
Sub-RT × task	0.126	0.868	0.75	0.943	0.141	0.652	0.182	0.136	0.333	0.75	0.438	0.085
Switches	0.317	0.889	0.873	0.798	0.247	0.216	0.225	0.129	0.625	0.376	0.602	0.751
Switches × task	0.127	0.45	0.519	0.561	0.136	0.081	0.167	0.032	0.415	0.219	0.829	0.85
WCP	0.204	0.973	0.794	0.647	0.569	0.767	0.622	0.532	0.01	0.291	0.778	0.634
WCP × task	0.526	0.413	0.743	0.199	0.993	0.33	0.799	0.778	0.128	0.206	0.231	0.504

Supplementary Data File S5

Behavior	Brain	estimate	p.value
CR	BA46_Left	-0.15909769	0.164
Sub_RT	BA46_Left	-0.18638324	0.102
Cluster_Size	BA46_Left	0.0097118685	0.933
Switches	BA46_Left	-0.08613129	0.453
WCP	BA46_Left	0.0273544045	0.813
BCP	BA46_Left	0.0207766916	0.856
CR	BA46_Right	-0.05842155	0.611
Sub_RT	BA46_Right	-0.14169375	0.215
Cluster_Size	BA46_Right	0.0039833835	0.972
Switches	BA46_Right	-0.00589333	0.959
WCP	BA46_Right	0.0550900092	0.634
BCP	BA46_Right	-0.01377104	0.905
CR	BA9_Left	-0.02297078	0.842
Sub_RT	BA9_Left	0.0197650450	0.863
Cluster_Size	BA9_Left	0.1131533855	0.324
Switches	BA9_Left	-0.09559863	0.405
WCP	BA9_Left	0.0007098211	0.995
BCP	BA9_Left	0.0161989908	0.888
CR	BA9_Right	-0.04350892	0.705
Sub_RT	BA9_Right	-0.14609441	0.201
Cluster_Size	BA9_Right	0.0359516176	0.755
Switches	BA9_Right	-0.03153247	0.784
WCP	BA9_Right	-0.00637525	0.956
BCP	BA9_Right	-1.26E-05	1
CR	IFG_Left	-0.17394697	0.128
Sub_RT	IFG_Left	-0.1730295	0.13
Cluster_Size	IFG_Left	0.0382404825	0.74
Switches	IFG_Left	-0.09525644	0.407
WCP	IFG_Left	0.0458623341	0.692
BCP	IFG_Left	0.0766701652	0.504
CR	IFG_Right	-0.01440583	0.9
Sub_RT	IFG_Right	-0.18575096	0.103
Cluster_Size	IFG_Right	0.0861043140	0.454
Switches	IFG_Right	-0.05554936	0.629
WCP	IFG_Right	0.0907519503	0.432
BCP	IFG_Right	0.1127100747	0.325
CR	MTG_Left	-0.18590749	0.103
Sub_RT	MTG_Left	0.0064113102	0.956
Cluster_Size	MTG_Left	-0.0240647	0.834
Switches	MTG_Left	-0.11804397	0.303
WCP	MTG_Left	0.2434686594	0.033

BCP	MTG_Left	0.2096005260	0.066
CR	MTG_Right	0.0420138507	0.715
Sub_RT	MTG_Right	0.0169830169	0.882
Cluster_Size	MTG_Right	0.1625346965	0.155
Switches	MTG_Right	0.0107093786	0.926
WCP	MTG_Right	0.1211559569	0.294
BCP	MTG_Right	0.1089164000	0.342
CR	OFC_Left	-0.06084153	0.597
Sub_RT	OFC_Left	-0.09156666	0.424
Cluster_Size	OFC_Left	-0.06140734	0.593
Switches	OFC_Left	-0.08440765	0.462
WCP	OFC_Left	-0.16553292	0.15
BCP	OFC_Left	0.1803892310	0.114
CR	OFC_Right	-0.10954516	0.34
Sub_RT	OFC_Right	0.0962202354	0.401
Cluster_Size	OFC_Right	-0.09112464	0.428
Switches	OFC_Right	-0.07260832	0.528
WCP	OFC_Right	-0.0176798	0.879
BCP	OFC_Right	0.2610174635	0.021
CR	SMG_Left	0.0196132210	0.865
Sub_RT	SMG_Left	0.2582101442	0.023
Cluster_Size	SMG_Left	-0.11788286	0.304
Switches	SMG_Left	0.0249801009	0.828
WCP	SMG_Left	0.1361410703	0.238
BCP	SMG_Left	0.3174420516	0.005
CR	SMG_Right	0.1243564369	0.278
Sub_RT	SMG_Right	0.2761415799	0.015
Cluster_Size	SMG_Right	0.0939193333	0.413
Switches	SMG_Right	0.0629888897	0.584
WCP	SMG_Right	0.0374890733	0.746
BCP	SMG_Right	0.0160725350	0.889

No effects survived FDR correction.

Supplementary Data File S6

p-values for the main effects of executive function test scores and their interaction with Condition on HbR activation levels across ROIs. No effects survived FDR correction.

	BA46_L	BA46_R	BA9_L	BA9_R	IFG_L	IFG_R	MTG_L	MTG_R	OFC_L	OFC_R	SMG_L	SMG_R
2back_accuracy	0.734	0.611	0.615	0.853	0.755	0.815	0.182	0.295	0.829	0.597	0.44	0.236
2back_accuracy × task	0.341	0.922	0.942	0.85	0.25	0.865	0.594	0.283	0.761	0.892	0.468	0.178
shiftdifference	0.163	0.833	0.159	0.966	0.363	0.622	0.754	0.709	0.571	0.814	0.963	0.984
shiftdifference × task	0.373	0.068	0.67	0.277	0.752	0.718	0.984	0.425	0.559	0.189	0.566	0.707
stroopratio	0.111	0.925	0.174	0.69	0.348	0.759	0.139	0.554	0.871	0.56	0.503	0.657
stroopratio × task	0.876	0.712	0.326	0.39	0.591	0.504	0.806	0.891	0.739	0.937	0.97	0.543
